# Transient and Steady-State Properties of *Drosophila* Sensory Neurons Coding Noxious Cold Temperature

**DOI:** 10.3389/fncel.2022.831803

**Published:** 2022-07-25

**Authors:** Natalia Maksymchuk, Akira Sakurai, Daniel N. Cox, Gennady Cymbalyuk

**Affiliations:** ^1^Neuroscience Institute, Georgia State University, Atlanta, GA, United States; ^2^Department of Physics and Astronomy, Georgia State University, Atlanta, GA, United States

**Keywords:** thermal sensation, computational modeling, *Drosophila* larvae, cold nociception, rate of temperature change, cold temperature magnitude

## Abstract

Coding noxious cold signals, such as the magnitude and rate of temperature change, play essential roles in the survival of organisms. We combined electrophysiological and computational neuroscience methods to investigate the neural dynamics of *Drosophila* larva cold-sensing Class III (CIII) neurons. In response to a fast temperature change (–2 to –6°C/s) from room temperature to noxious cold, the CIII neurons exhibited a pronounced peak of a spiking rate with subsequent relaxation to a steady-state spiking. The magnitude of the peak was higher for a higher rate of temperature decrease, while slow temperature decrease (–0.1°C/s) evoked no distinct peak of the spiking rate. The rate of the steady-state spiking depended on the magnitude of the final temperature and was higher at lower temperatures. For each neuron, we characterized this dependence by estimating the temperature of the half activation of the spiking rate by curve fitting neuron’s spiking rate responses to a Boltzmann function. We found that neurons had a temperature of the half activation distributed over a wide temperature range. We also found that CIII neurons responded to decrease rather than increase in temperature. There was a significant difference in spiking activity between fast and slow returns from noxious cold to room temperature: The CIII neurons usually stopped activity abruptly in the case of the fast return and continued spiking for some time in the case of the slow return. We developed a biophysical model of CIII neurons using a generalized description of transient receptor potential (TRP) current kinetics with temperature-dependent activation and Ca^2+^-dependent inactivation. This model recapitulated the key features of the spiking rate responses found in experiments and suggested mechanisms explaining the transient and steady-state activity of the CIII neurons at different cold temperatures and rates of their decrease and increase. We conclude that CIII neurons encode at least three types of cold sensory information: the rate of temperature decrease by a peak of the firing rate, the magnitude of cold temperature by the rate of steady spiking activity, and direction of temperature change by spiking activity augmentation or suppression corresponding to temperature decrease and increase, respectively.

## Introduction

Perception of cold pain plays an essential role in the survival of animals. It alarms them about harmful environments and navigates their escape into safe surroundings by employing remarkably precise and fast sensory mechanisms. The value of temperature in the cold range, the rate of temperature change, and duration of thermal stimulus are critical characteristics for health and survival (Altner and Loftus, 1985; Broman and Kallskog, 1995; Cheung et al., 2003; Gallio et al., 2011).

In mammals and insects, specialized cold-sensitive neurons have been found to encode either the actual temperature (Craig et al., 2001; Frank et al., 2015; Nagel and Kleineidam, 2015; Alpert et al., 2020) or the rate of temperature decrease (Loftus, 1968; Tichy, 1979; Ameismeier and Loftus, 1988; Gingl et al., 2005; Tichy et al., 2008; Ruchty et al., 2010; Nagel and Kleineidam, 2015; Luo et al., 2017), or both (Loftus, 1968; Merivee et al., 2003; Nishikawa et al., 2004; Gingl et al., 2005; Tichy et al., 2008; Olivares et al., 2015). Such neurons usually respond to temperature transition from higher to a lower steady level by a characteristic change in the spiking rate. The rate first increases to a certain maximum (peak), and then decreases and stabilizes at a new level, exceeding that observed before cooling. The initial transient and subsequent steady components of the spiking frequency response are also called phasic and tonic. The phasic component is characterized by a peak of the spiking rate during temperature change: the steeper the temperature drop, the higher the peak (Nishikawa et al., 1985; Merivee et al., 2003; Gingl et al., 2005; Must et al., 2006; Nagel and Kleineidam, 2015). The deeper the cooling level, the faster the tonic spiking (Davis and Sokolove, 1975; Merivee et al., 2003; Ruchty et al., 2009, 2010). In the cited works, the cooling was mostly innocuous, not deeper than 15^o^C. Hence, mechanisms that underlie sensing noxious cold temperatures remained largely unexplored.

*Drosophila melanogaster* is a powerful model organism for studying noxious cold sensation. It offers a variety of technical approaches to single cell-type manipulations. *Drosophila* has a relatively compact nervous system, and yet a rich repertoire of modality-specific sensory-induced behaviors as compared to mammalian systems (Pandey and Nichols, 2011; Turner et al., 2016; Jovanic, 2020; Himmel et al., 2021). *Drosophila* larvae have primary cold nociceptors, Class III (CIII) somatosensory neurons. In addition, Class II (CII) and chordotonal (Ch) neurons are also involved in noxious cold sensation (Turner et al., 2016, 2018; Himmel et al., 2021). *Drosophila* larval CIII primary afferents are activated by cold stimuli and mediate a stereotypic cold-evoked response behavior, a full-body contraction (CT) (Turner et al., 2016; Himmel et al., 2021).

Previous studies have demonstrated that molecular mechanisms responsible for temperature detection and signal transduction are conserved across various species (McKemy, 2007). Mammalian primary afferents express TRPA1 and TRPM8 channels, which are activated by cold (Jordt et al., 2003). Some non-TRP ion channels have been implicated in creating the appropriate conditions for cold-evoked electrical activity patterns: Kv1.2/1.2, TREK1/TRAAK, HCN, Na_*v*_1.9, T-type Ca_*v*_ (Orio et al., 2009; Latorre et al., 2011; Lolignier et al., 2016; Xu et al., 2017). *Drosophila* larva CIII neurons exhibit enriched expression of specific TRP channels: Trpm, Pkd2, NompC, and TRPA1, of which Trpm, Pkd2, and NompC have been demonstrated to participate in cold-evoked behavior (Turner et al., 2016, 2018), as well as enriched expression of Ca^2+^-activated potassium channels, which may contribute to the activity pattern of sensory neurons in response to noxious cold temperature (Turner et al., 2016). We instructed our model development with transcriptomic data on the specific ion channels expressed in CIII neurons; this information on relative expression levels of channels has been proved fruitful in the development of biophysically adequate computational models of other identified neurons (Baro et al., 1997; Schulz et al., 2006; Temporal et al., 2014; Tripathy et al., 2017; Bomkamp et al., 2019).

In vertebrates and invertebrates, TRP channels, such as TRPA1 and TRPM8, had been implicated in the coding of the rate of temperature change (Olivares et al., 2015; Luo et al., 2017). It has been previously shown that some TRP channels have Ca^2+^-dependent inactivation/desensitization, which leads to adaptation of cell response (Rohacs et al., 2005; Gordon-Shaag et al., 2008; McKemy, 2013). Moreover, the temporal scale for TRP channel desensitization is of a similar order as an adaptation of cold-sensitive neurons (Latorre et al., 2011; Olivares et al., 2015). Considering this basis, we developed a computational model that includes a generalized TRP current with temperature activation and Ca^2+^-dependent inactivation.

To investigate cellular mechanisms of noxious cold temperature coding, we combined electrophysiological experiments and computational modeling. We investigated temporal activity patterns of CIII neurons at different temperature protocols: steady innocuous and noxious temperatures and fast-changing thermal stimuli. Also, we explain our experimental finding with computational modeling, providing mechanisms based on the kinetics of TRP channels. Our study provides the first detailed experimental analyses of the cold-temperature-evoked responses and the first computational model of CIII primary sensory neurons. Therefore, it brings new insight into fundamental principles of neural coding of noxious and innocuous temperatures.

## Materials and Methods

### Animals

*Drosophila* stocks were maintained at 24°C under a 12:12 light:dark cycle. Age-matched wandering third-instar larvae were used for all electrophysiological experiments. In all the animals used, CIII neurons were identified by *GAL4-UAS*-mediated GFP labeling (*GAL4*^19–12^ > *UAS-mCD8::GFP*) (Turner et al., 2016).

### Electrophysiological Recordings

#### Dissection and Electrophysiology

The ventral midline of the third instar larva was incised, and all internal organs were removed by gentle pipetting. This “filet” preparation was pinned in a Petri dish lined with Sylgard^®^ 184 (Dow Corning) filled with HL3 saline (Stewart et al., 1994). Then, the body wall muscles were carefully removed with a tungsten needle and fine forceps. The dish was constantly superfused with gravity-dripped HL3 saline.

**FIGURE 1:**
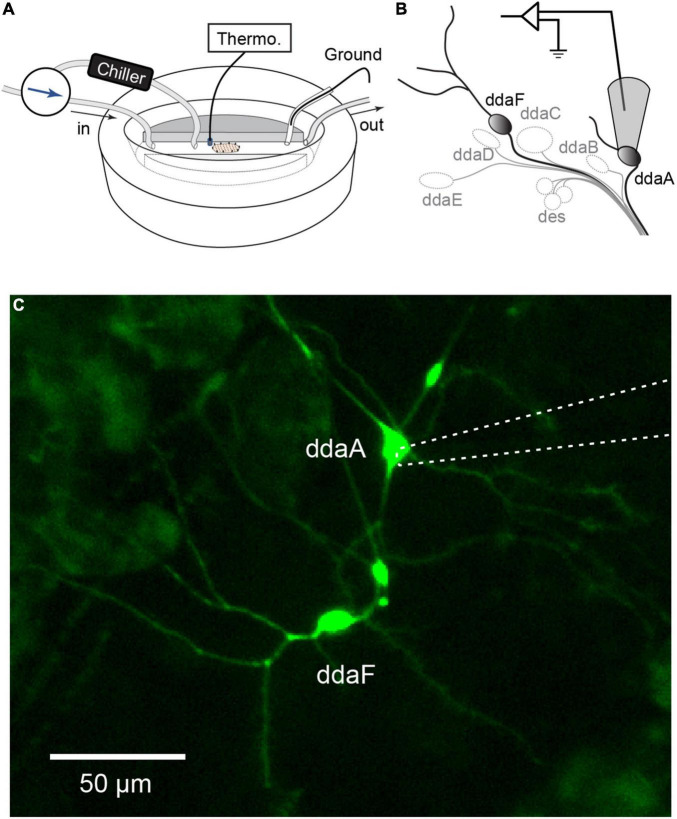
Extracellular recordings of CIII neurons in a *Drosophila* larval filet. **(A,B)** The larva filet was placed in an experimental chamber with running HL3 saline. To produce a controlled temperature decrease during the stimulus, the superfusion path was switched to the one that goes through the chiller, and chilled saline was delivered. The saline temperature was constantly monitored by a thermometer probe. The saline was grounded with an Ag-AgCl wire in an agar bridge. We recorded spiking activities from two subtypes of CIII neurons, ddaA or ddaF, located in the dorsal cluster of sensory neurons. To do this, the cell body with a portion of its neurite was gently sucked up into the pipette. **(C)** Image of CIII neurons (ddaA and ddaF) labeled by *GAL4*^19–12^, *UAS-mCD8::GFP*, with the electrode (a dotted line) attached to ddaA.

Extracellular recordings were made with a pipette (tip diameter, 5–10 μm) connected to the headstage of a patch-clamp amplifier (AxoPatch200B or MultiClamp 700A, Molecular Devices, San Jose, CA, United States) set to a voltage-clamp mode. All recordings were made from either ddaA or ddaF in the dorsal cluster of the peripheral sensory neurons (Figure 1). Gentle suction was applied to draw the soma and a small portion of the neurite of ddaA or ddaF into the pipette. The amplifier’s output was digitized at 10 kHz using a Micro1401 A/D converter (Cambridge Electronic Design, Cambridge, United Kingdom) and acquired into a laptop computer running Windows 10 (Microsoft, Redmont, WA, United States) with the Spike2 software v. 8 (Cambridge Electronic Design, Cambridge, United Kingdom). Saline temperature was continuously recorded by BAT-12 Microprobe Thermometer (Physitemp, Clifton, NJ, United States). The temperature probe was placed adjacent to the filet preparation (Figure 1A), and the readings were sent to Micro1401.

#### Cold Temperature Stimulation

Cold temperature stimulation was applied by passing saline through an in-line solution cooler (SC-20, Warner Instruments, Hamden, CT, United States) connected to the controller device (CL-100, Warner Instruments, Hamden, CT, United States) (Figure 1). To apply a fast temperature change, the superfusion path was quickly switched to run saline through SC-20 for the time of stimulation. To apply a slow temperature change, a command ramp waveform was created by the acquisition software Spike2 and was fed to the controller CL-100.

#### Cold Stimulation

We performed three types of cold temperature stimulation: (1) fast-stimulation protocol where fast temperature change was followed by an interval of time where the temperature was held constant; (2) slow-stimulation protocol where the temperature was slowly decreased to target temperature and held constant for 30 s, and then slowly raised back to the room temperature; and (3) step-stimulation protocol where temperature decreased with small steps to the target temperature. For the fast-stimulation protocol (1), the saline was chilled to 20, 15, or 10°C in advance by an SC-20 in-line solution cooler, and quick delivery of chilled saline was made by switching the superfusion path to the one that goes through SC-20 for 60 s. With this protocol, the temperature of the saline in the dish can be decreased at a rate of –2 to –6°C/s. The stimulus was ended by switching the superfusion path back. For the slow-stimulation protocol (2), the saline was chilled to 10^o^C at the rate of –0.12°C/s by being passed through SC-20. For the step-stimulation protocol (3), the solution temperature was changed in steps of –2.5°C until it reached 10^o^C, and the temperature was kept constant for at least 30 s at each step. In the slow-(2) and step-stimulation protocols (3), the temperature of SC-20 was set by a voltage command generated by the Spike2 software.

#### Spike Count and Statistics

**FIGURE 2:**
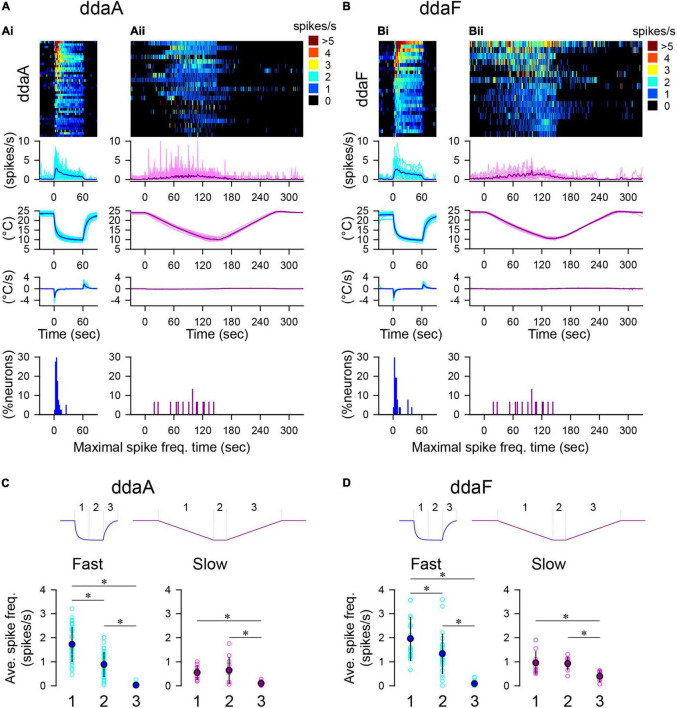
Spiking responses of CIII neurons to two types of temperature stimulation protocols. **(A,B)** Spiking activities of ddaA **(A)** and ddaF **(B)** in response to the fast-stimulation protocol (10°C, **Ai,Bi**) and the slow-stimulation protocol **(Aii,Bii)**. The panels show, from top to bottom, the heat map representation of the spiking rate, the plot of the averaged spiking rate against time, the temperature change, the rate of temperature change, and % histogram of the time bins (2 s), with the maximum spiking rate. In **(A,B)**, cyan and pink traces show individual data; blue and purple traces show the mean values. *N* = 40 **(Ai)**, 21 **(Aii)**, 26 **(Bi)**, and 15 **(Bii)**. Time zero is set to the onset of the stimulation. **(C,D)** The average spiking rate in ddaA **(C)** and ddaF **(D)** during the falling phase (1), the steady phase (2), and the rising phase (3) of the fast and slow stimulation protocols drawn schematically above the graphs. Asterisks indicate significant differences (see text).

**FIGURE 3:**
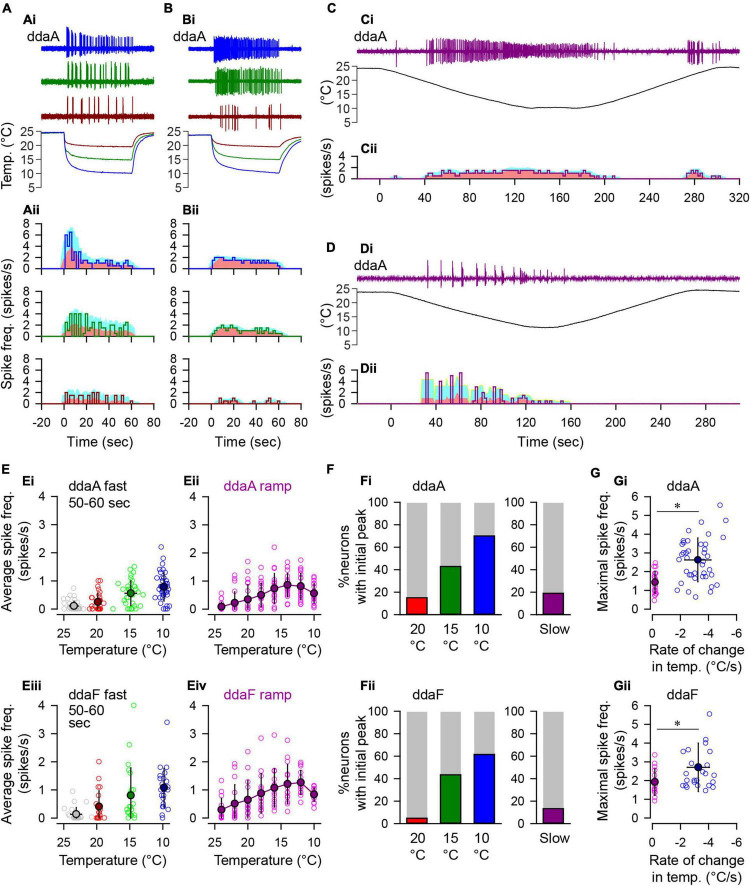
Spiking responses of CIII neurons to fast and slow temperature stimulation. **(A,B)** Two types of spiking responses to the fast-stimulation protocol. In **(Ai,Bi)**, three representative traces of spiking activity recorded extracellularly from two ddaA neurons are shown with corresponding temperature traces on the bottom **(Ai,Bi)**. The temperature was reduced to 20°C (dark red), 15°C (dark green), and 10°C (blue). Graphs in **(Aii,Bii)** show changes in the spiking rate (spikes/s, a solid line) in every 2-s bin. In each graph, the pink area indicates the running average of the spiking rate measured with a 10-s moving window, whereas the cyan area indicates standard deviation. **(C,D)** Two types of spiking responses to the slow-stimulation protocol. As in **(A,B)**, the spike traces with corresponding temperature traces **(Ci,Di)** and the changes in the spiking rate **(Cii,Dii)** are shown. **(E)** The relationship between the steady-state spiking rate vs. temperature. In **(Ei)** (ddaA) and **(Eiii)** (ddaF), the spiking rate in time windows of 50-60 s after the onset of the fast stimulation protocol is plotted against temperature. Symbols are color-coded based on the target temperature: 20°C, red/dark red; 15°C, green/dark green; and 10°C, blue/dark blue; room temperature, gray. In **(Eii)** (ddaA) and **(Eiv)** (ddaF), average spike frequencies (spikes/s) were measured in every 2°C bin from 25°C to 9°C during the slow ramp stimulation protocol. In all graphs, opened circles show individual data, and color-filled black circles show the averages. Error bars indicate standard deviations. **(F)** Bar graphs showing the percentage of neurons that exhibited a distinct peak within 20 s after the onset of the fast-stimulation protocol (20, 15, 10°C) and during the downslope (120 s) of the slow-stimulation protocol (Slow). **(G)** The maximal spike frequencies were plotted against the rate of change of temperature in the fast- (blue/dark blue) and slow-stimulation (pink/purple) protocols (**Gi**, ddaA; **Gii**, ddaF). The maximal spiking rate was measured by a 10-s running window that runs through all 1-s bins. The asterisks indicate *p* < 0.05 by *t*-test.

Spikes were detected by setting the amplitude threshold in the Spike2 software function, and their spiking rate (spikes/s) was calculated as an average over a fixed time window of 2 s (Figures 2A,B), 10 s (Figures 3Ei,iii), or as a moving average over a time window of 10 s (Figures 3A–D). To determine the relationship between a spiking rate and temperature in the slow stimulation protocol, we plotted the average of the spiking rate in a 2°C temperature bin (Figures 3Eii, iv). To determine whether there was a peak spike response in the fast-stimulation protocol (Figure 3F), we first determined the spiking rate per second, and then divided the stimulation period into six 10-s windows and compared the average spiking rate (spikes/s) in each time bin. If the average spiking rate was the highest in any of the first two bins among the six, we further examined whether the changes in the average spiking rate in the subsequent bins were significant by the one-way RM ANOVA. If both conditions were met, the spike response was determined to have a peak.

A decay time constant of the spiking rate was obtained by plotting the instantaneous spiking rate and fit decay phase by a double-exponential curve:


$$f\left(t\right)=ae^{-t/\tau 1}+ce^{-t/\tau 2},$$

where *t* is the time, *a* and *c* are steady-state spiking rates, and τ1 and τ2 are the decay time constant.

The temperature of half activation of the spiking rate was measured by the fast-stimulation protocol with three different temperatures as well as the slow and step-stimulation protocols. In the fast- and step-stimulation protocols, the average steady-state spiking rate was measured in the last 20 s when the temperature was held at a steady level. In the slow stimulation protocol, the average spiking rate was measured in every 2°C temperature bin from 25 to 9°C and plotted. These plots were fit by a sigmoidal curve:


(1) $$f\left(T\right)=\frac{a}{1+e^{-S\left(T-T_{hf}\right)}},$$

where *T* is the temperature and *T*_*hf*_ is the temperature of half activation of the spiking rate, *a* is the maximal spiking rate, and *S* is the steepness of the curve. If the temperature-response relationship did not have a maximal plateau or a peak of spiking activity, then *a* was set to be the maximum spiking rate observed in tonic spikes in the recording.

### Computational Modeling

We developed a Hodgkin-Huxley-type computational model of the cold-sensitive CIII neuron. The model is based on ionic current suggested by transcriptomic data of ion channels’ expression in CIII neurons (Turner et al., 2016): voltage-gated Na^+^ current, *I*_*Na*_-encoded by the *para* gene; delayed rectifier potassium current, I_*K*_ encoded by the *shab* gene; Ca^2+^-activated potassium channels: *I*_*SK*_, small, and *I*_*BK*_, big conductance, encoded by *SK* and *slowpoke* genes, respectively; voltage-gated calcium current, *I*_*Ca*_; leak current, *I*_*L*_, and TRP current, *I*_*TRP*_.

Our computational model includes the Na^+^ current, which is based on the *para* gene. Unlike mammalians, this is the only *Drosophila* gene encoding the α-subunit of voltage-gated sodium channels. Alternative splicing of the *para* gene generates a wide variety of splice variants of this gene and a wide range of gating properties and voltage dependencies for *Drosophila* voltage-gated Na^+^ channels (Olson et al., 2008). Insects do not have orthologs of mammalian β-subunits for Na^+^ channels (Feng et al., 1995). However, it was found that insect TipE and TEH auxiliary subunits are functional analogs to mammalian β-subunits (Dong et al., 2014). Using transcriptomic data (Turner et al., 2016), we found that CIII neurons express these auxiliary subunits. They modulate both expression level and gating properties of Na^+^ channels (Catterall, 2000; Brackenbury and Isom, 2011).

*I*_*Na*_ represents the DmNav26 splice variant of the *para* gene co-expressed with the TipE auxiliary subunit. DmNav26 is one of the splice variants, which is present in both late embryonic stages and adult flies (Lin et al., 2009). We used available electrophysiological data on this splice variant expressed in *Xenopus* oocytes together with TipE (Hardie et al., 1991; Olson et al., 2008; Wang et al., 2013). We assume that voltage-gated sodium channels are required for the generation and propagation of action potentials in CIII neurons, as the knockdown of the *para* gene in CIII neurons significantly impaired cold-evoked nociceptive contraction behavior or blocked optogenetic-induced contraction behavior in the *Drosophila* larva (Turner et al., 2016).

For voltage-gated K^+^ currents, *I_*K*_*, the gating characteristics were obtained from the experimental literature using patch-clamp data (Hardie et al., 1991). Delayed rectifier K^+^ current encoded by the *Shab* gene gives rise to currents similar to Hodgkin-Huxley K^+^ delayed rectifier ones (Tsunoda and Salkoff, 1995). We included N-type voltage-gated Ca^2+^ current (VGCC), represented by the *cacophony* (*cac*) VGCC in *Drosophila* CIII neurons. N-type VGCCs have been implicated in the modulation of sensory signals and pain processing (Park and Luo, 2010; Adams and Berecki, 2013).

*Drosophila* larval CIII neurons express a suite of TRP channels, among which the following have been implicated in noxious cold-evoked behavior: Trpm, encoded by the *Trpm* gene; Pkd2, encoded by the *Pkd2* gene; and NompC, encoded by the *nompC* gene (Turner et al., 2016). There are currently no electrophysiological data available on the gating properties of these *Drosophila* TRP channels. In our model, we used a basic TRP current representation, I_*TRP*_, which represents all cold temperature-sensitive TRP currents lumped together. I_*TRP*_ is a nonspecific current with a reversal potential close to zero. As some TRP currents are activated by chilling, having sigmoidal dependence on temperature (Feng, 2014), we modeled temperature-dependent activation of our generalized TRP current by the Boltzmann function with temperatures of half activation and steepness adjusted to reproduce experimental data. Some mammalian thermosensitive TRP channels, such as TRPM8, TRPM4, TRPA1, and TRPV1, exhibit voltage dependence (Nilius et al., 2005, 2011), while mammalian PKD1 or PKD1/PKD2 complex has been shown to have an almost linear current-voltage (I-V) relationship (Vandorpe et al., 2001; Babich et al., 2004). Different isoforms of *Drosophila* TRPA1 currents have distinct current-voltage curves (Gu et al., 2019). For example, *Drosophila* TRPA1-A has weak voltage dependence in the physiological range of membrane potentials (Gu et al., 2019). *Drosophila* Pkd2 and NompC currents have modest dependence on voltage (Venglarik et al., 2004; Yan et al., 2013). In this study, we focused on the roles of temperature and intracellular Ca^2+^ dependencies and assumed that the conductance of I_*TRP*_ either did not depend on the membrane potential. Since TRP channels have Ca^2+^-dependent inactivation/desensitization (Rohacs et al., 2005; Gordon-Shaag et al., 2008; McKemy, 2013), we implemented Ca^2+^-dependent inactivation of I_*TRP*_, with time constants of activation and inactivation estimated from the experimental decaying phase of the CIII firing rate. To introduce the temperature effect on the non-TRP currents, in our model, we used Q_10_ scaling factors for the time constants of the maximal conductances ρ(*T*) = 3.^−(*T*−*T*_0_)/10^ and activation variables φ(*T*)=1.3^(*T*−*T*_0_)/10^, where *T* is a temperature in K; *To = 298K* is a reference temperature (Hille, 2001). We used temperature in K for parameters of our biophysical model, while, in figures for consistence with experimental data, modeling results were presented with the temperature reported in °C.

Simulations and data analyses were implemented using custom-made MATLAB scripts (The MathWorks, Inc). To obtain a numerical solution of a system of differential equations, describing the model of CIII neuron, we used the variable-step integration method with backward differentiation formulas, with the absolute tolerance 10^–9^ and relative tolerance 10^–8^, using MATLAB function ode15s.

Our CIII model consists of spike-generating, fast subsystem: voltage-gated Na^+^ current, I_Na_, voltage-gated K^+^ current I_K_, leak current I_L_; pattern-generating, moderately slow subsystem: small conductance Ca^2+^-activated potassium current, I_SK_, big conductance Ca^2+^-activated potassium current, I_BK_, and the voltage-gated N-type calcium channel, I_Ca_; and thermotransduction subsystem: nonspecific TRP current, I_TRP._ We assume that temperature drop initiates depolarization of the cell by TRP current, and then spike-generating subsystem produces action potentials. The pattern-generating subsystem is responsible for the corresponding modality-specific pattern of CIII activity. The dynamics of the electrical activity of the CIII model are described by the equation:


$$\frac{dV_{m}}{dt}=-\left[I_{Na}+I_{K}+I_{Ca}+I_{BK}+I_{SK}+I_{L}+I_{TRP}\right]/C_{m},$$

where V_m_ is a membrane potential, C_m_ is a total neural membrane capacitance, C_m_ = 0.01 nF. The currents in the model are described by the following equations:


INa=ρ(T)⋅GNa¯⋅mNa3⋅hNa⋅[Vm-ENa],

IK=ρ(T)⋅GK¯⋅mK4⋅[Vm-EK],

$$I_{Ca}=\rho \left(T\right)\cdot \bar{G_{Ca}}\cdot m_{Ca}\cdot h_{Ca}\cdot \left[V_{m}-E_{Ca}\right]$$

IBK=ρ(T)⋅GBK¯⋅fCaBK⋅mBK4⋅[Vm-EK]

$$I_{SK}=\rho \left(T\right)\cdot \bar{G_{SK}}\cdot m_{SK}\cdot \left[V_{m}-E_{K}\right]$$

$$I_{L}=\rho \left(T\right)\cdot G_{L}\cdot \left[V_{m}-E_{L}\right];$$

The thermotransduction mechanism is based on a TRP current, with temperature-dependent activation, *m_TRP_*, and Ca^2+^-dependent inactivation, *h*_*TRP*_:


$$I_{TRP}=\bar{G_{TRP}}\cdot m_{TRP}\cdot h_{TRP}\cdot \left[V_{m}-E_{TRP}\right],$$

where $\bar{G_{i}}-$ is the maximal conductance, ρ(*T*) – the temperature-dependent scaling factor, *E_i_* is the reversal potential, *m_i_* and *h_i_* are activation and inactivation gating variables of current *i*, with *i ϵ {Na, K, Ca, BK, SK, TRP}*.

The reversal potential for TRP current takes into account changes in the intracellular Ca^2+^ concentration and is calculated as: ETRP=PKEK*+PNaENa*+PCaECa*PK+PNa+PCa, where *P*_*K*_, *P*_*Na*_, and *P*_*Ca*_ are relative permeabilities for corresponding ions. Relative permeabilities for K^+^and Ca^2+^ were constant, 1 and 0.4, respectively, whereas permeability for Na^+^ was calculated, taking into account zero reversal potential for the taken reversal potentials: *P*_*Na*_ = −(*P*_*K*_*E*_*K*_ + *P*_*Ca*_*E*_*Ca*_)/*E*_*Na*_, where *E*_*K*_, *E*_*Na*_, and *E*_*Ca*_ are equal to –75, 65, 120 mV, correspondingly.

All gating variables are described with the following equation:


$$\frac{dy_{i}}{dt}=\frac{f_{\infty i}\left(V\right)-y_{i}}{\tau_{i}\left(V\right)},$$

where *y*_*∞i*_ is a steady-state activation for the current *i ϵ {Na, K, Ca, BK, SK, TRP}*.


$$f_{CaBK}\left(Ca_{i}\right)=\frac{1}{1+{\left(\frac{Ca_{BK}}{Ca_{i}}\right)}^{n_{BK}}};m_{BK\infty }\left(V\right)=\frac{1}{1+e^{\frac{-\left(V+28.3\right)}{30}}};$$

$$m_{SK\infty }\left(Ca_{i}\right)=\frac{1}{1+{\left(\frac{Ca_{SK}}{Ca_{i}}\right)}^{n_{SK}}};$$

$$\tau_{mBK}\left(V\right)=\frac{-0.1502}{1+e^{\frac{-\left(V+46\right)}{22.7}}}+0.1806;$$

$$\tau_{hNa}\left(V\right)=\left(\frac{4.5}{\text{cosh}\left(\frac{V+V_{hNa}}{3K_{hNa}}\right)}+0.75\right)/1000;$$

$$\tau_{mK}\left(V\right)=\left(\frac{5}{\text{cosh}\left(\frac{V+V_{mK}}{2K_{mK}}\right)}+0.75\right)/1000;$$

Ca^2+^ dynamics is described by the equation:


$$\frac{dCa_{i}}{dt}=-\varphi \left(\frac{I_{Ca}+I_{TRP_{Ca}}}{F\cdot z\cdot Vol}+k\cdot \left[Ca_{i}-Ca_{min}\right]\right),$$

where *I*_*TRP_Ca_*_ is a Ca^2+^ component of TRP current. It is calculated as


$$I_{TRP_{Ca}}=\bar{G_{TRP}}\cdot m_{TRP}\cdot h_{TRP}\cdot \frac{P_{Ca}}{P_{K}+P_{Na}+P_{Ca}}\left[V_{m}-E_{Ca}\right].$$

We applied the following expression for steady-state TRP activation: $m_{TRP\infty }\left(T\right)=\frac{B}{1+e^{-A\left(T-T_{h}\right)}}$,

where *A* – the steepness of temperature dependence of TRP activation, *B* – the activation scaling factor, *T*_*h*_ – the temperature of the half activation in Boltzmann function in K, *T* – the temperature in K.

Steady-state inactivation of TRP current is:


$$h_{TRP\infty }\left(Ca_{i}\right)=1-\frac{Ca_{i}^{N}}{Ca_{h}^{N}+Ca_{i}^{N}}$$

where *Ca*_*i*_ – intracellular Ca^2+^ concentration, *Ca*_*h*_ – the half inactivation Ca^2+^ concentration, *N* – the Hill coefficient. Cytosolic Ca^2+^ concentration is a pivotal variable in the process of TRP inactivation. This process includes a number of sub-processes and stages, which are not well understood and even less described quantitatively. We investigated phenomena and variables, which we hypothesize are critical or dominant. Increased cytosolic Ca^2+^ triggers downstream pathways that underlie desensitization of many TRP channels that can occur *via* kinases, phosphatases, phospholipases, or calmodulin (Gordon-Shaag et al., 2008; McKemy, 2013). Our model uses a general expression for TRP inactivation that reflects Ca^2+^-dependent modulation of TRP current that can be caused by these pathways.

The reversal potentials for the voltage-gated K^+^, leak, and Na^+^ currents are –75, –75, and 65 mV, respectively. The Ca^2+^ reversal potential dynamically changes in time and is calculated as the Nernst potential using *Ca*_*i*_ and assuming that the external Ca^2+^ concentration (*Ca*_*e*_) is constant and equal 2 mM: $E_{Ca}=1,000\frac{RT_{K}}{zF}log\frac{Ca_{e}}{Ca_{i}}$, where *R*, *F*, and *T_K_* are the gas constant, Faraday’s constant, and temperature in Kelvin, respectively.

We applied thermal stimulation to our model using the same temperature protocols as in the experimental recordings, using temperature traces recorded from the microprobe thermometer (2.2.3). In addition, we applied trapezoid stimulation temperature protocols with (1) different rates of temperature change and the same target cold temperature or (2) different target temperatures and the same rate. In the case (1), it was held at 24^o^C for 30 s and then decreased linearly with different rates (the rate was swept from 0.1 to 5.5^o^C/s with an increment of 0.1^o^C/s from trial to trial) to the same target value of 10^o^C, which was then kept constant for 30 s. After that, the temperature was linearly raised to 24^o^C, with the same rate of change and held constant for 30 s afterward. In the second case (2), the temperature was held at 24^o^C for 30 s and then linearly decreased to different values (the target temperature was swept from 20 to 6^o^C, with a decrement of 0.5^o^C from trial to trial), with the same rate of 3^o^C/s. After reaching the target value, it was kept constant for 30 s and then linearly increased to 24^o^C with the same rate. Afterward, the temperature was held at this level for 30 s.

In addition, we characterized steady-state regimes of the model’s activities. For this, we recorded the activity of the model at a constant temperature. The parameter value of the temperature was swept in the range from 24 to 10^o^C, with a decrement of 0.25^o^C. Integration was performed over 100 s, and then data were collected over additional 5 s, and the spiking rate was calculated.

To evaluate whether the variability of CIII cold-evoked responses could be caused to the variability of the biophysical parameters of a TRP current, we used the parameters estimated using the experimental data from multiple CIII neurons. The temperature of half activation of spiking rate *T*_*hf*_, the maximal spiking rate *a*, and the steepness of the curve *S* from temperature-response characteristics fitted with a sigmoidal curve (Equation 1, Supplementary Table S1) were used as parameters for the TRP current: the temperature of the half activation in ^o^C *T*_*hC*_, the scaling factor for maximal TRP conductance *B*, the steepness of temperature dependence of TRP activation A, correspondingly. We considered two groups of the parameter sets where the Group I used parameter fits from the slow-stimulation protocol (*N* = 22), and the parameter sets of the Group II were based on the step-stimulation protocol (*N* = 27). In Table 1, the model has smaller numbers of instances N than experimental ones since the experimental protocols, which generated the fits required longer time and had a lower experimental success rate compared to the fast-stimulation protocols used to test responses of living and model neurons. One parameter set from the Group II was excluded from consideration since the model neuron incorporating it (G2-23, Supplementary Table S1) did not exhibit any spiking activity. We investigated spiking activity of the model using parameter sets from these two groups in response to actual experimental temperature traces of the fast-stimulation protocol, with the temperature decreasing down to 10°C. We also considered small variability in experimental temperature stimulation traces from experiment to experiment; we applied to every parameter set the corresponding experimental temperature trace of the same protocol. We distinguished responses with a detected phasic component and report percentage of such responses. In addition to the steady-state spiking rate, for such responses, we reported the peak value of the spiking rate. We also determined coefficients of variation for these three measurements, taking into account two factors causing variability of responses: variability of TRP parameter sets and variability in temperature stimulation (Table 1). Detection of the phasic component and evaluation of the peaks in the model spiking responses were performed using the one-way RM ANOVA in the same way as in the analysis of experimental responses.

**TABLE 1:** Coefficients of variation (CoVs) of responses (for peak and steady-state spiking rates) of model neurons with two groups of the parameter sets and living CIII neurons (pooled ddaA and ddaF data).

CoV of	Model Group I	Model Group II	Experiment
Peak spiking rate	0.53 (*N* = 15)	0.59 (*N* = 12)	0.39 (*N* = 44)
Steady-state, responses with peak	0.49 (*N* = 15)	0.41 (*N* = 12)	0.73 (*N* = 44)
Steady-state, responses without peak	0.58 (*N* = 7)	0.51 (*N* = 14)	0.65 (*N* = 22)

*The parameter sets for the models in Group I and Group II were obtained by curve fitting the spiking rates of CIII neurons induced by the slow-stimulation protocol and by step-stimulation protocol, respectively. The responses are induced by experimental fast-stimulation protocols with an end temperature of 10^o^C.*

In the model, we also separately considered two factors that could cause variability of responses between neurons: variability of TRP parameter sets and variability in temperature stimulation. As in the previous case, we investigated model responses based on parameter sets from Group I and Group II. We determined coefficients of variations of peak and steady-state spiking rates for every fixed parameter set at different temperature traces (an NxN matrix, *N* = 22 for Group I and *N* = 26 for Group II). Also, we calculated model responses for every fixed temperature trace to different parameter sets (an NxN matrix, *N* = 22 for Group I and *N* = 26 for Group II). Obtained results are presented in Supplementary Table S2.

The model parameters are the following: $\bar{G_{NaF}}=80nS$, $\bar{G_{K}}=140nS$, $\bar{G_{Ca}}=3.5nS$, $\bar{G_{BK}}$ = 6 nS, $\bar{G_{SK}}$ = 0.31 nS, *G*_*L*_=0.28*nS*, *n*_*BK*_=3, *Ca*_*BK*_ = 1,700 nM, *Ca*_*SK*_=800*nM*, *n*_*SK*_=3, *V*_*mNa*_ = −24.7*mV*, *K*_*mNa*_=3.4*mV*, *V*_*hNa*_ = −41.2*mV*, *k*_*hNa*_=4.2*mV*, *V*_*mK*_ = −12*mV*, *K*_*mK*_=7*mV*, *V*_*mCa*_ = −23*mV*, *K*_*mCa*_=6.5*mV*, *V*_*hCa*_ = −59*mV*, *K*_*hCa*_=15*mV*, F = 96,485.35 10^–9^ C/nM, R = 8.31 10^–9^J/(nM*K), C_m_ = *.01 nF, z = 2, Vol = 0.2 pL, k = 403 s^–1^*, *Ca*_*min*_ = *50 nM*, [Ca^2+^]_e_ = *2 10^6^ nM*, τ_*mSK*_ = 0.04*s*, τ_*mK*_=0.0025*s*,τ_*mCa*_=0.0035*s*,τ_*hCa*_=0.095*s*,τ_*Na*_ = 0.0001 *s, B = 1.* Canonical parameters for TRP current are: $\bar{G_{TRP}}$ *= 1.2 nS; T_h_ = 290 K; A = 1K^–1^; N = 2; Ca_h_ = 700 nM;* τ_*hTRP*_ = *10 s;* τ_*mTRP*_= 0.002 s.

## Results

### CIII Neurons Have a Phasic-Tonic Response to Temperature Decrease

We performed detailed electrophysiological recordings of CIII neurons in response to cold temperature stimulation (Figure 1). To investigate the activity of CIII neurons at steady cold temperature or in response to changes of temperature, we used three different temperature stimulations: (1) fast-stimulation (2) slow-stimulation, and (3) step-stimulation protocols.

#### CIII Responds With a Peak of the Spiking Rate to Fast Temperature Change

At room temperature (22–24^o^C), the majority of CIII neurons were silent or exhibited spontaneous spiking with a rate of less than 1 Hz. The spontaneous spiking was seen in 60% of ddaA neurons (*N* = 24 of 40) and 73% of ddaF neurons (*N* = 19 of 26). On average, the spontaneous spiking rates were 0.11 ± 0.17 Hz (mean ± SD; *N* = 40) in ddaA and 0.13 ± 0.24 Hz (mean ± SD; *N* = 26) in ddaF.

To investigate the effect of a noxious cold temperature, we applied two different patterns of the cold-temperature stimulation (fast- and slow-stimulation protocols) that brought the saline temperature down to 10°C (Figure 2). In response to both the fast- and slow-stimulation protocols, all CIII neurons either started spiking or increased the rate of ongoing spiking activity (Figures 2A,B; *N* = 40 for ddaA and *N* = 26 for ddaF). On average, the spiking rate reached maximal levels shortly after the onset of a fast stimulation, which was followed by a slow decline to a steady state (Figures 2Ai,Bi). The times of the maximum spiking rate were found near the stimulus’s onset when the temperature was rapidly falling (percentage histograms in Figures 2Ai,Bi). In 85% of ddaA (*N* = 34 of 40) and 81% of ddaF (*N* = 21 of 26), the spiking rate reached the maximal point within the first 10 s of the stimulation interval and then gradually declined to a steady state.

In contrast to the fast-stimulation protocol, a slow-stimulation protocol caused a gradual increase of the spiking rate during the downslope of the temperature in the majority of neurons (ddaA, *N* = 18 of 21; ddaF, *N* = 14 of 15; Figures 2Aii,Bii). Some neurons, however, showed notable fluctuations over time (cf. Figure 3D). Because of this, the timing of the maximum spiking rate varied from cell to cell (percentage histograms in Figures 2Aii,Bii). The average times of the maximal spiking rate in the slow-stimulation protocol are 63.9 ± 32.3 s for ddaA and 89.1 ± 36.9 s for ddaF (means ± SD), which are significantly larger than the fast-stimulation protocols of the same temperature (10°C; 6.8 ± 5.2 s for ddaA and 8.9 ± 9.7 s for ddaF; *p* < 0.05 for both ddaA and ddaF by Mann–Whitney rank-sum test). There was also a significant difference in variance between the fast and slow-stimulation protocols in both ddaA and ddaF (*p* < 0.05 by Levene median tests).

There is an asymmetry in the rate of spiking activity between the decrease and increase of the temperature change. The spiking rate during the downslope of temperature change was significantly higher than that during the upslope (Figures 2C,D). To compare the change in spiking activity with temperature change, we divided the temperature stimulation into three phases (1–3 in Figures 2C,D). In the fast stimulation protocol, the three phases were characterized by significantly different rates of spiking activity exhibited by the CIII neurons (*p* < 0.001 by one-way RM ANOVA with the Holm–Sidak multiple comparisons, *N* = 40 for ddaA, *N* = 26 for ddaF; graphs on the left in Figures 2C,D). In the slow-stimulation protocol, there was a significant decrease in the spiking rate in Phase 3 in both ddaA and ddaF (*p* < 0.001 by one-way RM ANOVA with Holm–Sidak multiple comparisons, *N* = 11 for ddaA, *N* = 10 for ddaF), but no significant difference in the mean spiking rate between the downslope Phase 1 and the constant-temperature Phase 2 (graphs on the right in Figures 2C,D).

The average spiking rate in Phase 1 of the fast stimulation protocol was 1.72 ± 0.70 spikes/s (mean ± SD, *N* = 40) for ddaA and 1.96 ± 0.89 spikes/s (mean ± SD, *N* = 26) for ddaF, which were both significantly higher than those in the same phase of the slow-stimulation protocol (ddaA, 0.55 ± 0.29 spikes/s, *N* = 11; ddaF, 0.96 ± 0.45 spikes/s, *N* = 10; *p* < 0.001 for both by Mann–Whitney Rank Sum Test). In Phase 3, when the temperature was returning back to room temperature, most of the CIII neurons became silent within 2 s in the fast stimulation protocol (*N* = 38 of 40 for ddaA, *N* = 18 of 26 for ddaF), whereas, in the slow-stimulation protocol, most of them continued spiking for more than 10 s after the temperature started to rise (*N* = 9 of 11 for ddaA, *N* = 16 of 21 for ddaF). The spiking rate in Phase 3 of the slow-stimulation protocol was significantly higher than that in fast-protocol Phase 3 (ddaA, 0.021 ± 0.045 spikes/s in the fast protocol and 0.10 ± 0.08 spikes/s in the slow-stimulation protocol; ddaF, 0.090 ± 0.092 spikes/s in the fast protocol and 0.40 ± 0.023 spikes/s in the slow-stimulation protocol *p* < 0.05 by the Mann–Whitney Rank Sum test). There was no significant difference in the spiking rate in Phase 2 between the two protocols (*p* > 0.05 by the Student’s *T*-test) in both ddaA and ddaF. Thus, these results show that, when the amplitude of the temperature change was the same, a fast temperature decrease caused a higher spiking rate of response, and a fast temperature increase caused a faster cessation of spike activity.

#### Steady-State Activity of CIII Neurons Grows With a Magnitude of Cold Temperature

To assess the dependence of the firing rate on the magnitude of cold stimulation, we applied the fast stimulation protocol with three different target temperatures (10, 15, and 20°C; Figures 3A,B), and compared them with the responses to slow stimulation protocol (Figures 3C,D).

In the fast-stimulation protocol, all of the tested cells responded by spiking to 10 and 15°C stimuli (10°C, ddaA *N* = 40, ddaF *N* = 26; 15°C, ddaA *N* = 35, ddaF *N* = 23), but 20°C stimuli evoked no response in some cells (ddaA, *N* = 5 of 33; ddaF, *N* = 2 of 21). CIII neurons often showed an initial peak in spike frequency (Figure 3Aii). The number of neurons with an initial peak response increased with the increasing magnitude of the fast cold stimulus (Figure 3F). In response to the 10°C fast stimulation protocol, 70% of ddaA (*N* = 28 of 40) and 61.5% ddaF (*N* = 16 of 26) exhibited spiking activities with a pronounced initial spiking rate peak and a subsequent frequency adaptation when the temperature reached a stationary level (Figure 3A). To 15°C stimuli, 42.9% of ddaA (*N* = 15 of 35) and 43.5% of ddaF (*N* = 10 of 23) exhibited a peak; to 20°C stimuli, 15.2% of ddaA (*N* = 5 of 33) and 4.8% of ddaF (*N* = 1 of 21) exhibited a peak. On the other hand, a small number of neurons maintained a nearly constant spiking rate without showing an initial peak (10°C, ddaA, *N* = 12 of 40; ddaF, *N* = 10 of 26), which increased with milder temperature stimulation (to 15°C stimuli, 20 of 35 ddaA and 13 of 23 ddaF; to 20°C, *N* = 28 of 33 ddaA and *N* = 20 of 21 ddaF; Figure 3B). The average spiking rates at the peak (measured by 10-s bins) were 2.78 ± 1.03 spikes/s (CoV = 0.37, *N* = 28) in ddaA and 2.73 ± 1.16 spikes/s (CoV = 0.43, *N* = 16) in ddaF in response to the fast 10°C stimulation. There was no significant difference between ddaA and ddaF in the peak spiking rate (*p* > 0.05 by the Student’s *T*-test). Among the neurons that exhibited a peak spiking rate, the average spiking rates at the steady state (50–60 s after the onset of the stimulus) were 0.76 ± 0.50 spikes/s (CoV = 0.65, *N* = 28) in ddaA and 0.99 ± 0.78 spikes/s (CoV = 0.79, *N* = 16) in ddaF. Among those without the peak, the average steady-state spiking rates were 0.83 ± 0.53 (CoV = 0.63, *N* = 12) spikes/s in ddaA and 1.44 ± 0.81 (CoV = 0.56, *N* = 10) spikes/s in ddaF. There was no statistical difference between the peaked and non-peaked responses in the mean value of the steady-state spiking rate in both ddaA and ddaF (*p* > 0.05 by Student’s *T*-test). The steady-state spiking rate in the fast-stimulation protocol increased at the lower temperature, i.e., for the higher magnitude of the cold stimulation (Figures 3Ei,iii; *p* < 0.001 by the Kruskal–Wallis one-way ANOVA, *N* = 33–40 for ddaA, *N* = 21–26 for ddaF). The magnitude of spiking responses was highly variable among cells, ranging from 7 to 52 spikes in the first 10 s and 0 to 34 spikes in the last 10 s of 60-s stimulation.

In the slow-stimulation protocol, most neurons showed spiking at a relatively constant spiking rate or a gradual increase in the spiking rate, with a decrease in the temperature (Figure 3C), while only a few cells showed an initial peak response (ddaA, *N* = 3 of 21; ddaF, *N* = 1 of 15; Figures 3D,F). The average spiking rate reached maximal value before reaching 10°C in both CIII neuron subtypes (Figures 3Eii,iv). Between the fast- and slow-stimulation protocols of the same stimulus magnitude, there was a significant difference in the maximal spiking rate, with the fast protocol eliciting responses with significantly higher maximal spiking rates in both ddaA and ddaF (Figure 3G; Student’s *T*-test, *p* < 0.05). Altogether, these results suggest that CIII neurons are responsive to both the magnitude and the rate of temperature change.

**FIGURE 4:**
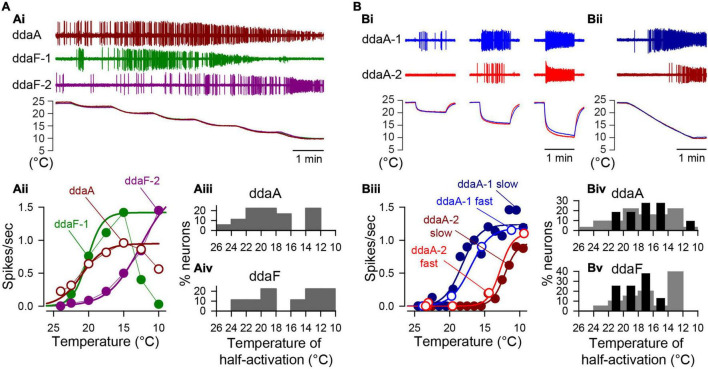
The cold temperature sensitivity of individual CIII neurons was evaluated by curve fitting their steady-state spiking rate to the Boltzmann function of steady temperature value. **(A)** Examples of spiking activity of three different CIII neurons (one ddaA and two ddaFs) obtained with the step-stimulation protocol, showing different threshold temperatures. One neuron (ddaF-1) completely stopped spiking at the lowest temperature step (10°C). The activities, along with temperature recordings, are shown **(Ai)**. The plots of the average spiking rate corresponding to each temperature **(Aii)**, and percentile histograms of temperature of half activation for the spiking rate **(Aiii,Aiv)** are presented. **(B)** Two contrasting examples of spiking activities of two ddaA neurons in response to the fast-stimulation protocol reaching three different steady temperatures **(Bi)** and to the slow-stimulation protocol **(Bii)** are shown. In **(Bi,Bii),** the upper traces (blue and dark blue) and the lower traces (red and dark red) were recorded from the same set of cells, ddaA-1 and ddaA-2, respectively. The plots of spiking rates (spikes/s) against temperature **(Biii),** and percentile histograms of temperature of half activation for the spiking rate **(Biv,Bv)** were also shown. In the fast protocol **(Bi)**, the average spike rate was measured during a 20-s window near the end of each stimulus. In the slow ramp protocol **(Bii)**, the average spiking rate was measured for every 2°C bin (16–17 s). In **(Biv,Bv)** the gray bars show data by the fast-stimulation protocol, whereas the black bars by the slow-stimulation protocol.

To evaluate the dependence of the spiking rate on the cold temperature value, we estimated the temperature of half activation of the spiking rate in each neuron by three different stimulation protocols: the fast-stimulation protocol and the slow-stimulation protocol, as used in Figures 2, 3, and the step-stimulation protocol (Figure 4Ai). In the step- and fast-stimulation protocols, the steady-state activity of CIII neurons was measured in the last 20-s window when the temperature was kept constant (Figures 4Ai,Bi). In the slow-stimulation protocol, the spiking rate was measured in every 2°C bin from 25 to 9°C during the temperature changing at the rate of –0.12°C/s (Figure 4Bii; see the section “Materials and Methods”).

In all neurons examined by the step-stimulation protocol, spike discharges were observed as the temperature decreased (ddaA, *N* = 18; ddaF, *N* = 9). There was a remarkable neuron-to-neuron variation of the maximal spiking rate and the temperature at which the maximal activity occurred (Figures 4Ai,ii). There were two types of responses; the proportion of which differed between ddaA and ddaF neurons. Of ddaA neurons, 61% exhibited a maximum of the spiking rate at a certain temperature, and the rate declined with lower temperatures (*N* = 11 of 18), whereas 39% (*N* = 7 of 18) exhibited a sigmoidal increase of the spiking rate along with the decreasing temperature (Figure 4Aii). In contrast, 78% of ddaF neurons showed a sigmoidal increase of the spiking rate, with the temperature decreasing (*N* = 7 of 9), and 22% had a maximum at the certain temperature higher than 10°C (*N* = 2 of 9). Currently, it is unknown whether this decline was due to the temperature-dependent change or the time-dependent spike-frequency adaptation. We determined the temperatures of half activation of the spiking rate by curve fitting of minimal to maximal spiking responses to a sigmoidal curve based on the Boltzmann function (see section “Materials and Methods”). The temperature of half activation of the spiking rate varied from cell to cell, ranging from 26 to 10°C in both ddaA and ddaF (Figures 4Aiii,iv). There was no statistical difference in average temperature of half activation of the spiking rate between ddaA and ddaF (ddaA, 18.5 ± 3.9°C; ddaF, 16.0 ± 4.3°C; *p* = 0.14 by Student’s *T*-test).

Notable neuron-to-neuron variation was also found between the responses evoked by either the fast- or slow-stimulation protocols. In the examples shown in Figure 4B, two ddaA neurons showed distinct temperature-response relationships; however, each ddaA showed similar response curves to the two stimulation protocols. One neuron (ddaA-1) showed a robust spiking activity at milder temperature (around 15°C) in both the fast- and slow-stimulation protocols (Figures 4Bi–iii, blue and dark blue plots), whereas the other (ddaA-2) showed less activity at the same temperature range but reached the maximal spiking rate at a lower temperature (Figures 4Bi–iii, red and dark red plots). Similar variation was seen in ddaF neurons (not shown); altogether, there was no significant difference between the average temperature of half activation of the spiking rate between ddaA and ddaF in either protocol (the fast-stimulation protocol, *p* = 0.40 by the Mann–Whitney Rank Sum Test, *N* = 24 for ddaA and 20 for ddaF; the slow-stimulation protocol, *p* = 0.20 by Student’s *T*-test, *N* = 9 for ddaA and 10 for ddaF). However, the temperature range was wider in the fast stimulation protocol than in the slow-stimulation protocol (Figures 4Biv,v); the temperatures of half activation of the spiking rate for individual neurons distributed from 26 to 10°C in the fast-stimulation protocol and from 24 to 14°C in the slow-stimulation protocol. These results suggest that individual cells were tuned to distinct temperature ranges of their own. The majority of ddaA and ddaF neurons (72.2% in ddaA, *N* = 26 of 36; 81.8% of ddaF, *N* = 18 of 22) exhibited a sigmoidal increase of the spiking rate with the maximal response at 10°C in the fast-stimulation protocol. The rest of the neurons had a peak at 15°C, and the spiking declined at 10°C (data not shown). With the slow-stimulation protocol, fewer cells showed sigmoidal responses, 27.3% in ddaA (*N* = 3 of 11) and 60% in ddaF (*N* = 6 of 10), and the rest exhibited the maximum in their temperature dependences.

Together, the results demonstrate that: (1) CIII neurons strongly responded to temperature change, with a pronounced peak of the firing rate and subsequent frequency adaptation. (2) Steady-state spiking rates of CIII neurons were temperature dependent. The spiking rate grew with decreasing steady temperature. (3) There was high variability in the temperature of half activation of the spiking rate across individual CIII neurons, which may indicate that individual cells are tuned to distinct temperature ranges. The results suggest that a single neuron does not respond evenly to the entire temperature range, but, rather, that each neuron responds to a different temperature range, thus encoding the temperature collectively as a population. Similar diversity in sensitivity of the spiking rate to temperature changes has been observed in other insects (Loftus, 1968; Merivee et al., 2003).

**FIGURE 5:**
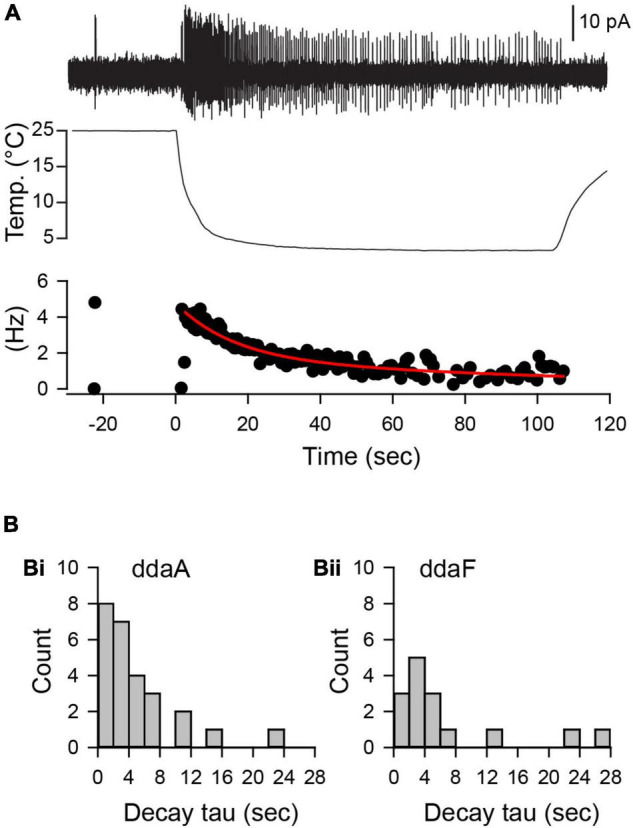
The decline of the spiking rate during the noxious cold stimulation showed an exponential decay with a broad distribution of time constants of decay. **(A)** A representative spiking response (a top trace) of a ddaA neuron to a 10°C fast-temperature stimulation (middle traces). A plot of instantaneous spike frequencies (Hz) was shown at the bottom. **(B)** Histograms of decay time constant in ddaA **(Bi)** and ddaF **(Bii)** in response to a 10°C temperature stimulation.

To characterize dynamic responses of CIII neurons to a fast-changing cold stimulus, we evaluated time constants of spiking rate decay after it reaches the peak of the spiking rate and then declines to a steady level. The spiking rate decay with time can be fitted by a double exponential curve with an initial fast decay and a slower decay later during the stimulation to obtain characteristic time constants (Figure 5). The mean decay time constant of the spiking rate in ddaA was 5. ± 5.1 s (mean ± SD, *N* = 26), ranging from 1.2 to 23.8 s, whereas that of ddaF was 6.7 ± 7.9 s (mean ± SD, *N* = 15), ranging from 0.8 s to 27 s. Despite faster decay times for ddaA neurons compared to ddaF ones, there was no statistical difference in the decay time constants between ddaA and ddaF (*p* = 0.60 by the Mann–Whitney Rank Sum Test).

### Basic Kinetics of a Model TRP Current Offer a Mechanism Explaining Phasic-Tonic Responses of CIII Neurons

**FIGURE 6:**
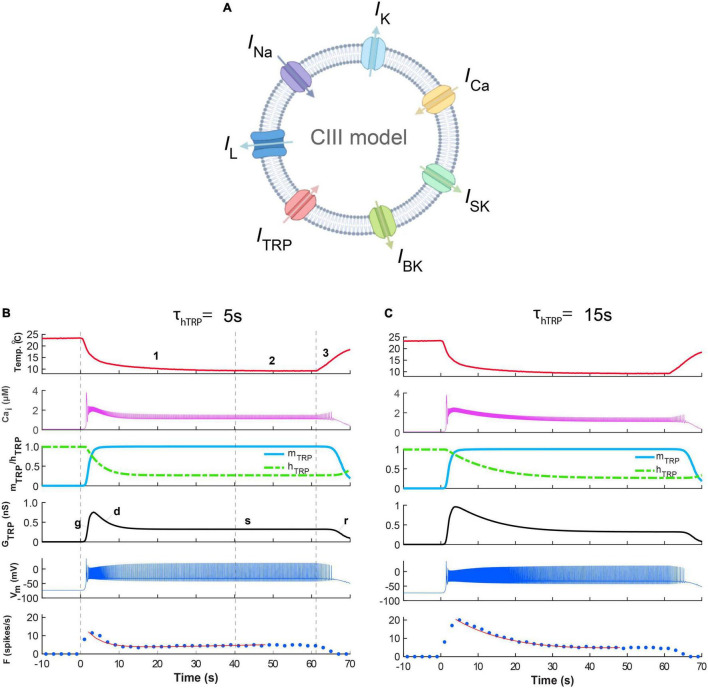
Responses of the CIII model reflect three phases of the fast-stimulation temperature protocol. The difference in the time constants of the Ca^2+^-dependent inactivation of TRP current produces the difference between the time course of the spiking rate decay. **(A)** The CIII computational model includes voltage-gated Na^+^ current, *I*_*Na*_, encoded by the *para* gene*; voltage-gated K^+^* current *I_*K*_*, encoded by the *Shab* gene; the voltage-gated N-type calcium channel, *I*_*Ca*_, encoded by the *cacophony* gene; leak current *I_*L*_;* and TRP current, *I*_*TRP*_; small conductance Ca^2+^-activated potassium current, *I*_*SK*_, encoded by the *SK* gene; and big conductance Ca^2+^-activated potassium current, and *I*_*BK*_, encoded by the *slowpoke* gene. In **(B,C),** the fast-stimulation protocol and model parameters are the same, except for the time constant of inactivation of the TRP current: τ_*hTRP*_ = 5 s **(B)** and τ_*hTRP*_ = 15 s **(C)**. **(B,C)** The experimental temperature trace (the top panel, red), intracellular Ca^2+^ concentration (the second panel, magenta), activation and inactivation of the TRP current (the third panel, blue and green, respectively), TRP conductance (the fourth panel, black), cold-evoked spiking activity response (the fifth panel, navy blue), and the spiking rate (a 2-s bin, bottom, navy blue) for the model with observed fast **(B)** and slow **(C)** spiking rate decays after the peak. In the bottom panel, the red line is a curve fitting the firing rate, with a double exponential function. The parameters of TRP current shared between the model in **(B,C)** are $\bar{G_{TRP}}$ = 1.2 nS, *T*_*h*_ = 290 K (16.85^o^C), A = 1 K^–1^, *N* = 2, Ca_h_ = 700 nM, τ_*mTRP*_ = 0.002 s. Letters “g,” “d,” “s,” “r” show phases of TRP conductance, *G*_*TRP*_: g, growing; d, declining; s, steady, and r, returning of *G*_*TRP*_ to its initial level.

We hypothesize that responses of CIII neurons to transient and steady noxious cold stimuli could be determined by TRP channels’ kinetics, which represents a thermo-transduction mechanism. First, we applied the fast-stimulation temperature protocol to our model (Figure 6). Two presented examples (Figures 6B,C) exhibited the characteristic phasic-tonic responses with the peak of the spiking rate during the temperature drop interval, followed by the decay of the spike rate down to a steady-state level. These responses were in correspondence with the spiking responses to the same fast-stimulation protocol in the electrophysiological experiments (Figures 2Ai,Bi, 5). The models presented in B and C (Figure 6) differ in one parameter – the time constant of TRP inactivation, τ_*hTRP*_. This difference was reflected in fast (Figure 6B) and slow (Figure 6C) spiking rate decays corresponding to τ_*hTRP*_ = 5 s and τ_*hTRP*_ = 15 s, respectively. We curve-fitted these two traces of the spiking rate by a double exponential function in the same way as in the experimental data analysis, and obtained estimations for the decay time constants as 3.9 s and 15.8 s for B and C, respectively, that were consistent with experimental decay time constants (Figures 5Bi,ii). These curve-fitted values, 3.9 and 15.8 s, were close to the actual model values of τ_*hTRP*_, 5 and 15 s, that suggests that the spiking rate decay time constant could be used to roughly estimate the inactivation time constants of TRP channels.

Gated by the dynamics of its activation and inactivation, the response of TRP conductance to temperature (Figures 6B,C) reflected three phases of the temperature stimulation (Figures 2C,D): (1) decrease, (2) steady phase, and (3) increase. The decrease phase induced a peak of conductance formed by the quick temperature-controlled raise of the activation variable (Phase g, Figures 6B,C), followed by a slower process of Ca^2+^-governed decrease of the inactivation variable. The increase of the TRP conductance caused membrane depolarization, leading to the rapid growth of the spiking rate (Figures 6B,C). This process increased intracellular Ca^2+^ concentration (Phase g, Figures 6B,C) due to large Ca^2+^ influx carried by the voltage-dependent Ca^2+^ current and by the notable Ca^2+^ component of the TRP current. In turn, the raised Ca^2+^ concentration inactivated the TRP conductance. Near the intersection of the activation and inactivation traces (Phase g, Figures 6B,C), there was a peak of *G*_*TRP*_, which underlays the peak of the spiking rate, since the spiking rate roughly followed the TRP conductance, *G*_*TRP*_. The second phase (d, Figures 6B,C) was described by the decrease of *G*_*TRP*_ and the spiking rate. This suggested that the magnitude of the *G*_*TRP*_ peak determined the timing and the magnitude of the peak of the spiking rate. The steady phase s (Figures 6B,C) reflected the interval with roughly constant low temperature (Phase 2 of the temperature protocol) by the spiking rate relaxed down to its steady-state level. Then, at the return, Phase r, which corresponded to Phase 3 of the temperature protocol, the temperature returned to the basal level, and the spiking rate quickly diminished. All these changes in the model spiking rate with temperature were consistent with the experimental results (Figures 2Ai,Bi).

Thus, our CIII model reproduces the basic features of spiking responses of CIII neurons to fast-changing and steady cold temperatures. A rapid inactivation (∼3–20 s) of TRP currents could be responsible for the initial peak of the spiking rate at rapid temperature fall and subsequent spiking rate decay (spiking frequency adaptation) when the temperature reaches a steady level. In contrast, the spiking rate at the steady state could encode the absolute value of the temperature.

### The Dynamics of TRP Current Could Explain the Sensitivity of CIII Neurons to the Rate of Temperature Change and Cold Temperature Magnitude

**FIGURE 7:**
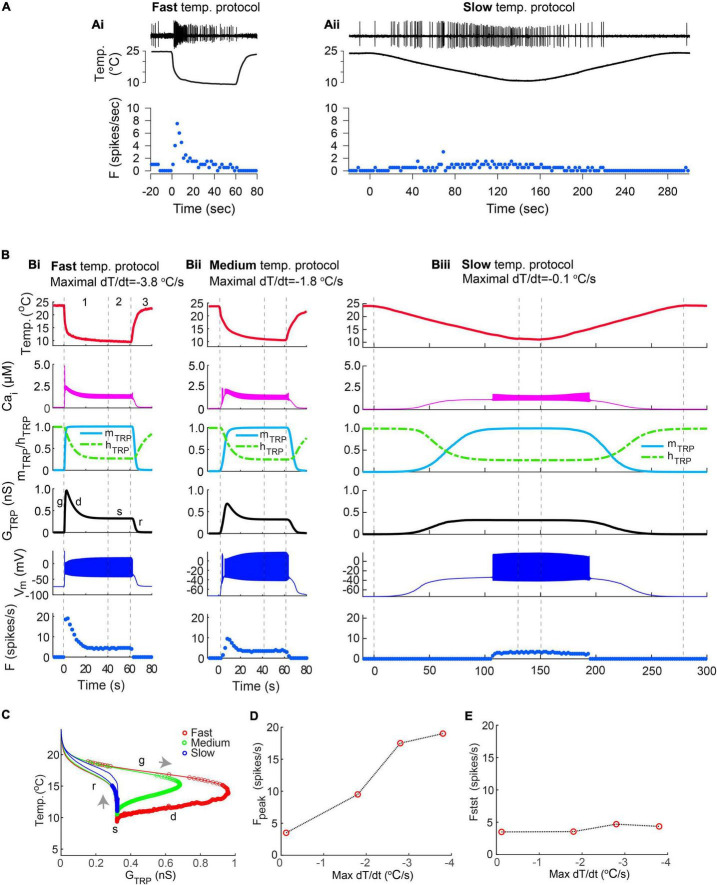
**(Ai,Aii)** Examples of CIII responses to the fast-stimulation protocol **(Ai)** and to a slow-stimulation protocol **(Aii)** obtained by electrophysiological experiments. (**Bi–iii**) A model reproduces the diversity of responses seen in electrophysiological recordings as being due to differences in the rate of temperature changes. Fast and medium temperature changes produced a well-defined peak of the spiking rate at the beginning of the temperature stimulus **(Bi,Bii)**, whereas slow temperature decrease did not produce any notable peak **(Biii)**. Numbers correspond to the phases of temperature protocol: 1 – temperature declines to the noxious cold range, 2 – steady noxious cold temperature, 3– temperature rises to an ambient level. The fast, medium, and slow rates of temperature decrease were –3.8°C/s in **(Bi)**, –1.8°C/s in **(Bii)**, and –.12°C/s in **(Biii)**, respectively. Letters “g,” “d,” “s,” “r” show phases of TRP conductance mentioned in Figure 6: g, growing; d, declining; s, steady, and r, returning of *G*_*TRP*_ to its initial level. **(C)** The faster rate of temperature decrease, the higher peak of conductance of TRP current, *G*_*TRP*_. Gray arrows indicate the direction of GTRP trajectory at temperature change: temperature decrease and increase correspondingly. Color circles indicate individual spikes. **(D,E)** Dependence of the maximal spiking rate **(D)** and a steady-state frequency **(E)** on the maximal rate of temperature change. The bin size for frequency is 2 s. Parameters of the model were $\bar{G_{TRP}}$ = *1.2 ns; T_h_ = 290 K; A = 1 K^–1^; N = 2; Ca_*h*_ = 700 nM;* τ_*hTRP*_ = *10 s;* τ_*mTRP*_ = *0.002 s.*

To investigate the effects of the rate of temperature decrease, we applied temperature stimulation protocols with different rates to our CIII model. These were the same fast- and slow-stimulation temperature protocols we used in experiments with live CIII neurons (Figures 7Ai,Aii). We chose the time constant of the TRP activation variable to be sufficiently faster than the rate of temperature change to follow either stimulation protocol. Thus, the activation variable assumed values in accordance with its steady-state temperature dependence [(*m*_*TRP*_(*T*) in Methods]. In such a way, the fast temperature decrease (the maximum temperature rate was –3.8^o^C/s) caused corresponding fast activation of TRP conductance, G_TRP_, up to the maximal value and a matching rapid rise of the spiking rate (Figure 7Bi). At the fast temperature drop, TRP activation and TRP conductance reached their maximal value before the temperature attained its minimal value at the steady phase. The Ca^2+^ fluxes carried by the TRP current and by the voltage-gated Ca^2+^ current raised intracellular Ca^2+^ concentration (Figure 7Bi); and a prominent rise of intracellular Ca^2+^ concentration was observed along with the fastest temperature change (Figure 7Bi). This led to Ca^2+^-dependent inactivation of the TRP current (Figure 7Bi), which determined the peak of *G*_*TRP*_ and the peak of the spiking rate (Figure 7Bi). A decrease in the rate of temperature change down to –1.8^o^C/s (Figure 7Bii) was reflected by a correspondingly slower rate of TRP activation (Figure 7Bi), which increased the delay to the maximal TRP activation. The peaks of the intracellular Ca^2+^ concentration, TRP conductance, and spiking rate (Figure 7Bii) were smaller than in Figure 7Bi due to Ca^2+^-dependent inactivation. Finally, at a slow temperature decrease, –0.1^o^C/s (Figure 7Biii), activation was accordingly slow (Figure 7Biii), inactivation followed corresponding intracellular Ca^2+^ concentration (Figure 7Biii), and, as a result, intracellular Ca^2+^ concentration, TRP conductance, and firing rate did not exhibit pronounced peaks (Figure 7Biii).

When the temperature was quickly returning from the noxious cold to room temperature (Phase 3 of the temperature protocol), spiking activity of the CIII model was suppressed. At a fast temperature rise, the firing rate rapidly declined to zero even if the temperature was still in the noxious cold range (Figure 7Bi). In contrast, when the temperature rose slowly (Figure 7Biii), the CIII neuron model continued to produce spiking activity even at relatively warm temperatures. Thus, similar to experimental data on CIII neurons, the CIII model asymmetrically responded to temperature change, reflecting the fast temperature decrease and its steady value and exhibiting suppressed or reduced spiking activity when temperature increased.

Trajectories of TRP conductance underwent different passages, depending on the rate of temperature change (Figure 7C): the faster the rate of temperature change, the faster the change of TRP current activation and the longer path of *G*_*TRP*_ trajectory and the higher peak of *G*_*TRP*_. We assessed the dependences of the peak of the spiking rate, *F*_*peak*,_ and steady-state frequency, *F*_*stst*_ on the maximal rate of temperature change; peaks grew along with the increase of the rate of temperature decrease (Figure 7D), whereas steady-state frequency was independent of the rate of temperature change (Figure 7E).

**FIGURE 8:**
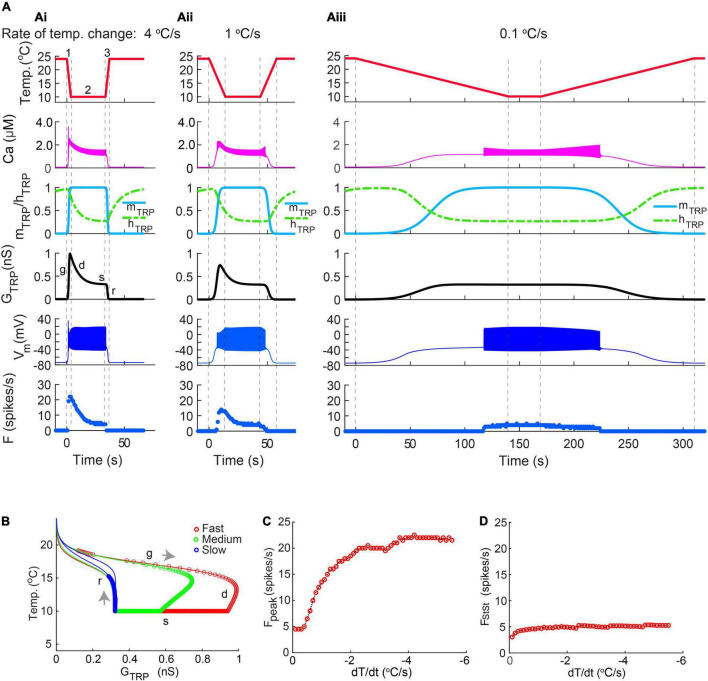
The CIII model responses to linear temperature change. Like exponential temperature change (Figure 7), the peak of the firing rate at trapezoid stimulation temperature protocol was determined by the rate of temperature change. **(A)** Fast temperature change induces a strong peak of the spiking rate at the beginning of the temperature stimulus **(Ai,Aii)**. Slow temperature change **(Aiii)** did not produce a peak of the spiking rate. The absolute value of the rate of temperature decrease and increase was 4^o^C/s in **(Ai)**, 1^o^C/s in **(Aii)**, and 0.1^o^C/s in **(Aiii)**. Numbers 1, 2, and 3 in **(Ai)** mark three phases of temperature protocol: (1) temperature decline, (2) steady temperature, and (3) temperature rise. **(B)** The higher rate of temperature decrease, the larger peak of conductance of TRP current, *G*_*TRP*_. Circles mark individual spikes. Arrows indicate the direction of temperature change: temperature decrease and increase correspondingly. **(C)** Dependence of the maximal spiking rate on the rate of temperature change. **(D)** The dependence steady-state spiking rate on the rate of temperature change. Bin size for frequency is equal to 2 s. Letters mark phases of TRP conductance changes (Figure 6): g, growing; d, declining; s, steady, and r, returning to the initial level. The parameters of the model are the same as in Figure 7.

In fast-stimulation protocols applied to live and model CIII neurons, the temperature decreased and increased exponentially, and the rate of temperature change, dT/dt, changed with time (Figures 7Ai,Bi,ii). To simplify the analysis of CIII model responses, we used a trapezoid stimulation protocol with a linear temperature change, and, thus, the rate of change was constant at the described intervals with the same rate of temperature change for both decrease and increase in temperature (Figure 8). Similar to the previous protocols in Figure 7, this protocol produced a peak of the spiking rate immediately after the fast temperature drop, which slowed down as the temperature decreased (Figures 8Ai,ii). At the fast rate of temperature change, –4^o^C/s, the temperature reached its final value, 10^o^C, so rapidly that TRP conductance, *G_*TRP*_*, and spiking rate did not reach the steady state within such a short time frame of temperature change (Figure 8Ai). With slower stimulations (–1 and –0.1^o^C/s), the CIII neuron model response had more time to reach a steady state (Figures 8Aii,iii). TRP conductance *G*_*TRP*_ underwent a longer path with faster rates of temperature change (Figure 8B). Flat s regions for dT/dt = 1^o^C/s and dT/dt = 4^o^C/s indicated that temperature reached the steady value faster than the TRP current gets inactivated (τ_*hTRP*_ = 10 s).

Similar to exponential fast-stimulation temperature protocols in Figure 7, the peak frequency increased with the rate of temperature change, and the steady-state spiking rate was not affected, Figures 8C,D, correspondingly. However, different dependencies of temperature over time, linear, and exponential brought discrepancies in the dependence of the spiking rate peak on the rate of temperature change. In the case of the trapezoid stimulation temperature protocol, it underwent saturation (Figure 8C), whereas, in the exponential fast-stimulation protocol (Figure 7D), it was roughly proportional to the rate of temperature change and does not give saturation at the same dT/dt range.

**FIGURE 9:**
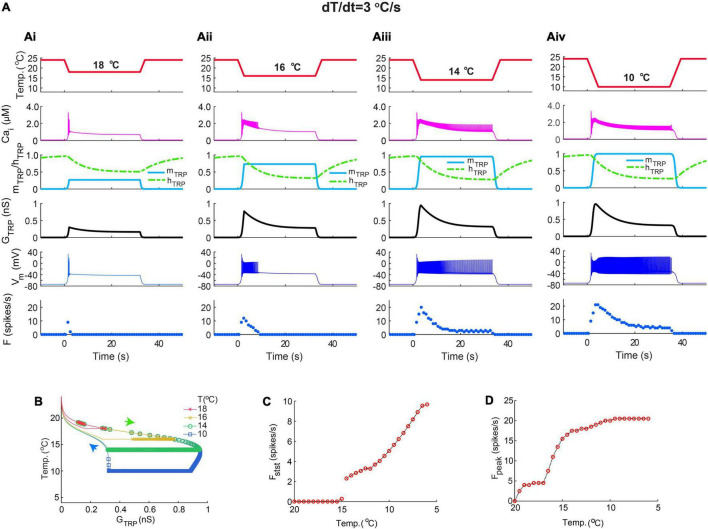
The CIII neuron model responds to the magnitude of steady cold temperature. **(Ai–Aiv)** Representative examples of the CIII model responses to different magnitudes of cold temperature at a fixed rate of temperature change, 3^o^C/s. **(B)** Trajectories of TRP current conductance at different cold temperature magnitudes are shown in **(Ai–Aiv)**. **(C,D)** Dependences of the spiking rate peak **(C)** and steady-state frequency **(D)** on the magnitude of the cold temperature value. **(B)** In size for the spiking rate is equal to 2 s. The parameters of the model are the same as in Figure 7.

Next, we evaluated responses to the magnitude of steady cold temperature at a fixed rate of temperature change, dT/dt = 3^o^C/s (Figure 9). Decrease of steady cold temperature caused an increase in intracellular Ca^2+^, TRP activation, m_TRP_, TRP conductance, G_TRP_, membrane depolarization, V_m_, and spiking rate (Figures 9Ai–iv). Colder temperatures elongated trajectories of TRP conductance and increased its maximal values (Figure 9B). The magnitude of cold temperature distinctly impacted frequency peaks and steady-state frequency, Figures 9C,D, correspondingly. Dependence of the frequency peak on cold temperature magnitude undergoes saturation at colder temperatures (Figure 9C), whereas steady-state frequency strongly increases with cold temperature magnitude (Figure 9D). All these data support the hypothesis that TRP current dynamics with temperature activation and Ca^2+^-dependent inactivation can explain not only the mechanism of coding the rate of temperature change but also cold temperature magnitude.

To evaluate whether the variability of experimental responses could be attributed to the variability of the biophysical parameters of TRP current, we tested experimentally obtained sets of parameters assessed by curve fitting of spiking rates (see methods) by introducing them in the model and applying experimental temperature protocols. In addition, we considered another factor that can cause variability – small variations in temperature stimulations that were applied to experimental neurons (see section “Materials and Methods”). We considered two groups of the sets, Group I had parameter fits from the slow-stimulation protocol (*N* = 22), and Group II was based on the step-stimulation protocol (*N* = 26). We found that 68% of Group I and 46% of Group II responses had a pronounced peak of the spiking rate at the fast temperature drop. These results are consistent with the percentage of biological CIII neurons: 67% of CIII neurons (70% of ddaA and 61.5% of ddaF) exhibited a peak in their responses. Thus, Group I matched and Group II had the same order of magnitude with this percentage. Representative model responses with and without peak of spiking rates that are based on different parameter sets are shown in Supplementary Figure S1.

These two groups of the model parameter sets produced models that showed similar variability in terms of coefficients of variation (CoV) of the peak and steady-state spiking rates. The variability of the model responses within the groups had the same orders of magnitude of CoVs as in corresponding experimental CoVs (Table 1), although the measurements of CoVs of Group I were about 10–15% closer to the experimental ones. These results suggest that the estimation of the parameters based on the curve fitting of the slow-stimulation protocol data might provide a better approximation than the step-stimulation protocol data. The slow-stimulation protocol might be more robust because it generates a larger amount of experimental data points for the curve fit. Concerning the model responses exhibiting the peak versus those which did not, there was no statistical difference between their steady-state spiking rates in either group (*p* > 0.05 by the Student’s *T*-test), which is consistent with the experimental results.

We also assessed variabilities caused by two factors separately: variability in parameters of TRP current and variability in temperature stimulation. Since there was no significant difference between steady-state spiking rates of responses exhibiting and not exhibiting the peak, we pooled these data together (Supplementary Table S2). Based on the results in Supplementary Table S2, we determined the mean CoVs for model responses (Table 2). We found that variations in parameters of TRP current had a strong effect on both the peak and the steady-state firing rate for both Groups I and II and had the same orders of magnitude as corresponding experimental CoVs (Table 2). This can tell us that individual variations between neurons of TRP current parameters can affect both characteristics of cold-evoked CIII response, the peak and steady state. Mean CoVs for the peak of the spiking rate induced by variation between the temperature stimulation traces were 60 and 68% smaller compared to mean CoVs from TRP current parameters variation for Group I and Group II correspondingly; however, variability in temperature stimulation still had a noticeable effect on the peak of the spiking rate (Table 2). Mean CoVs for the steady-state spiking rate due to temperature variation had relatively small effect on the steady-state firing rate, 88.7 and 76.6% smaller than CoVs due to parameters for Group I and Group II correspondingly.

**TABLE 2:** Coefficients of variation of model responses (for peak and steady-state spiking rates) with the two groups of the parameter sets (Group I and Group II in Table 1).

Mean CoV of	Model, Group I		Model, Group II		Experiment ddaA and ddaF
	Parameters variations	Temperature variations	Parameters variations	Temperature variations	
Peak spiking rate	0.6 (*N* = 484)	0.24 (*N* = 484)	0.72 (*N* = 676)	0.23 (*N* = 676)	0.39 (*N* = 44)
Steady-state spiking rate	0.53 (*N* = 484)	0.06 (*N* = 484)	0.45 (*N* = 676)	0.11 (*N* = 676)	0.71 (*N* = 66)

*The mean CoVs due to parameter variation were obtained by averaging CoVs of responses of each parameter set to all experimental temperature traces. The mean CoVs due to temperature variation were obtained by averaging CoVs of responses of all parameter sets to each experimental temperature trace.*

### Effect of TRP Current Parameters on the Peak and Steady-State Frequency

**FIGURE 10:**
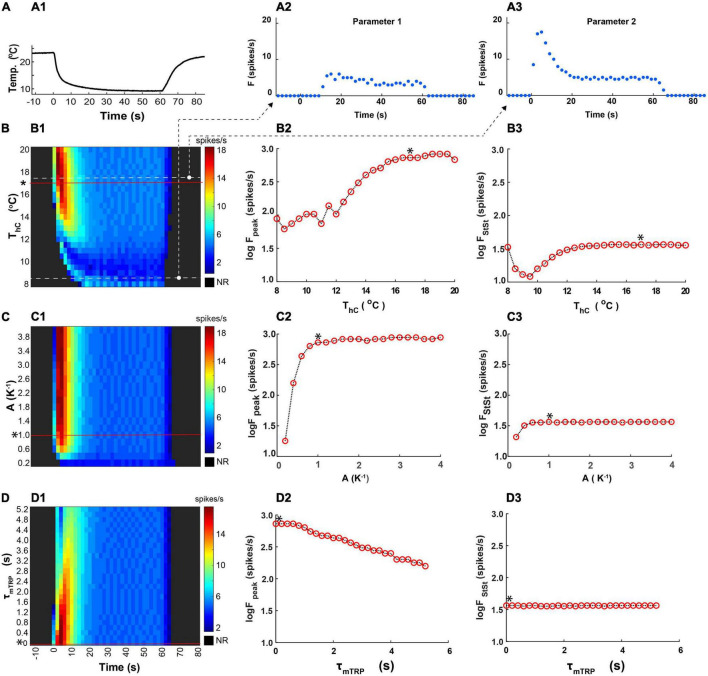
Effects of parameters determining kinetics of activation of the TRP current on the peak and the steady-state spiking rate of the CIII model’s responses to the fast temperature stimulation protocol. **(A1)** A representative example of the fast stimulation trace used in an experiment was applied to the CIII model. **(A2,A3)** Representative CIII model responses to this temperature protocol at different values of temperature of half activation: *T*_*h*_ = 281.5K and 290.5K, respectively. For convenience, Parameter T_h_ is represented by *T*_*hC*_ in ^o^C, 8.35^o^C, and 17.35^o^C, respectively. **(B1–D1)** Color maps code spiking responses (*x* axis) of the CIII model to the stimulation trace **(A1)** using different values of the parameter of interest (*y* axis) with other parameters kept at their canonical values* (a canonical parameter set in Figure 7). In all color maps, the average frequency was calculated in the 2-s bin window. **(B2–D2,B3–D3)** Dependences of the spiking rate peak and the steady-state spiking rate, respectively, on the parameter values: temperature of half activation, T_h_ (T_hC_ is in ^o^C, **B**), the steepness of TRP activation, A **(C)**, and time constant of TRP activation, τ_mTRP_ **(D)**. NR, no response.

To assess the sensitivity of the model responses to the variation of basic parameters, determining dynamics of the activation and inactivation of the TRP current, we investigated the effects of the parameter changes on the peak and the steady-state frequency of the CIII model response to fast temperature stimulation (Figures 10A1–A3). We changed one parameter at a time, keeping the rest of the parameters constant and equal to their canonical values (Figure 7).

The temperature of the half activation, T_h_, determines the threshold for TRP current activation. We applied the same fast-stimulation protocol (Figure 10A1) to the models with different *T*_*h*_ (*T*_*hC*_ is in ^o^C for convenience) (Figure 10B1). We compared model responses by representing them on a color map, where color describes the spiking rate changing in time along with column number. At small values of *T*_hC_, there was no apparent spiking rate peak, and the response was with a delay. With *T*_*hC*_ roughly above 13^o^C, the model exhibited a spiking rate peak. Then, with increasing *T*_*hC*_, the peak of the firing rate grew in magnitude (Figure 10B2). The temperature of half activation notably affected the spiking rate peak and did not affect the steady-state spiking rate for *T*_*hC*_ above 13^o^C (Figures 10B2,3). The dependence of steady-state frequency on *T*_*hC*_ had a minimum at *T*_*hC*_ near 9.5^o^C.

Similar analysis was applied to variation of the steepness of TRP steady-state activation, *A* and time constant of activation, τ_*mTRP*_. The steepness of TRP steady-state activation affected the peak and the steady-state spiking rate only at small values and did not affect them at larger values (Figures 10C1–3). The time constant of activation affected only the peak of the spiking rate and did not influence the steady-state spiking rate (Figures 10D1–3). Faster TRP activation (lower τ_mTRP_ value) produced a larger frequency peak during a temperature drop (Figures 10D1,2).

**FIGURE 11:**
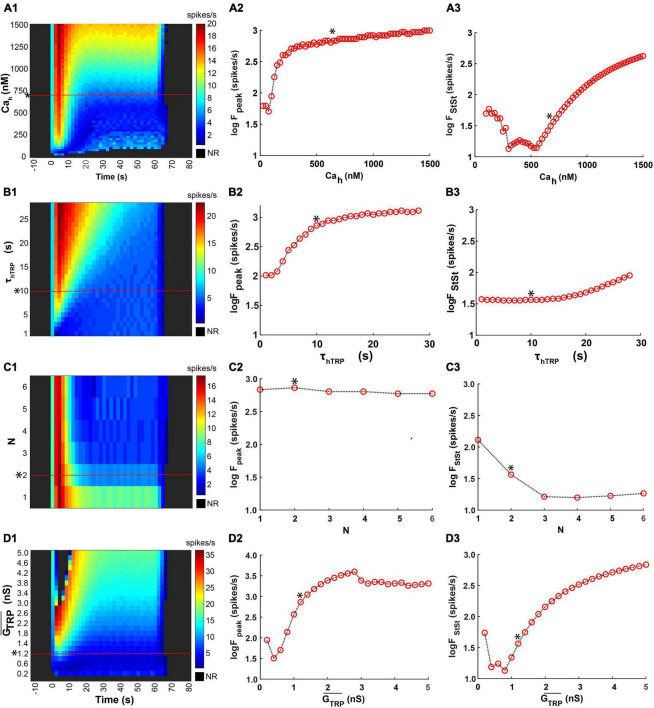
Effects of parameters of inactivation kinetics and maximal conductance of the TRP current on the model responses. We used here the same temperature stimulation trace (Figure 10A1), the canonical set of the model parameters, measurements of the responces, and the format of the color maps as in Figure 10. **(A1–D1).** We investigated effects of the Ca^2+^ concentration of half inactivation, *Ca*_*h*_ **(A1–A3)**, the time constant of TRP inactivation, τ_*hTRP*_ **(B1–B3)**, the Hill coefficient, *N* **(C1–C3)**, and the TRP current maximal conductance, $\bar{G_{TRP}}$, **(D1–D3)**.

In our model, Ca^2+^-dependent inactivation of the TRP current is governed by three major parameters: the Ca^2+^ concentration of half inactivation, *Ca*_*h*_, the time constant of inactivation, τ_*hTRP*_, and the Hill coefficient, *N*. At small values of *Ca*_*h*_, the model exhibited response only at the beginning of thermal stimulus, and then it turned silent despite an ongoing cold temperature stimulus (Figure 11A1, bottom rows). This happens because, at small *Ca*_*h*_ values, the TRP current is partially inactivated at room temperature. An increase of Ca_h_ resulted in an initial fast and then slow increase in the spiking rate peak (Figure 11A2). The steady-state spiking rate initially decreased at smaller values of *Ca*_*h*_ then, after certain Ca_h_, increased (Figure 11A3). Such character of the steady-state spiking dependence on *Ca*_*h*_ can be explained by different electrical activity regimes at different *Ca*_*h*_ values. The time constant of TRP inactivation, τ_*hTRP*_, also impacted the peak but not the steady-state spiking rate (Figure 11B1–B3). The larger τ_*hTRP*_ supported higher spiking rate peaks (Figure 11B2), whereas the steady-state spiking rate appeared to be independent of τ^*hTRP*^ up to 10 s and started to grow afterward (Figure 11B3). The Hill coefficient, *N*, describing the steepness of the TRP inactivation did not affect the spiking rate peak (Figures 11C1,2), but, in contrast, the steady-state frequency decreased as N increased from 1 to 3 and then stayed roughly constant (Figure 11C3).

Finally, we investigated the impact of maximal TRP conductance, $\bar{G_{TRP}}$on the peak and steady-state model response (Figure 11D1). Variation of $\bar{G_{TRP}}$ value could describe the effect of different levels of TRP expression in CIII neurons. At $\bar{G_{TRP}}=0$ nS, there was no model response to cold temperature (Figure 11D) since there were no other inward currents, except TRP current, in the model that is activated with a cold temperature. At small values of $\bar{G_{TRP}}$, the CIII model did not show a prominent peak of the spiking rate at rapid temperature change (Figure 11D1). The spiking rate peak grew with a larger $\bar{G_{TRP}}$ until spiking activity undergoes a depolarization block (a black cluster on a color map corresponding to rapid temperature decrease) (Figure 11D1). Because of the depolarization block, the spiking rate peak decreased at $\bar{G_{TRP}}$ > 3 nS (Figure 11D2). The steady-state spiking rate had a minimum at smaller $\bar{G_{TRP}}$ values and then rose with larger $\bar{G_{TRP}}$ values (Figure 11D3).

Thus, parameters describing TRP activation: *T_h_*, τ_*mTRP*_, *A* had a higher impact on the peak of the firing rate at a fast temperature drop, rather than on the steady-state rate when the temperature was constant. In turn, parameters describing TRP inactivation, *Ca*_*h*_ and *N*, had a stronger impact on the steady state than on the peak. Small values of the time constant of inactivation, τ_*hTRP*_ (τ_*hTRP*_ ≤ 10 s) significantly affected the peak of the firing rate, whereas larger values of this parameter (τ_*hTRP*_ 10 s) impacted the steady-state spiking rate. Both peak and steady-state spiking rates increased with increased $\bar{G_{TRP}}$. Our results suggest that variability in the parameters of TRP currents can explain the variability of the CIII neural responses to rapid temperature decrease and steady-state temperature.

### The CIII Neuron Model Explains Different Types of Experimental CIII’s Dose-Response Curves

**FIGURE 12:**
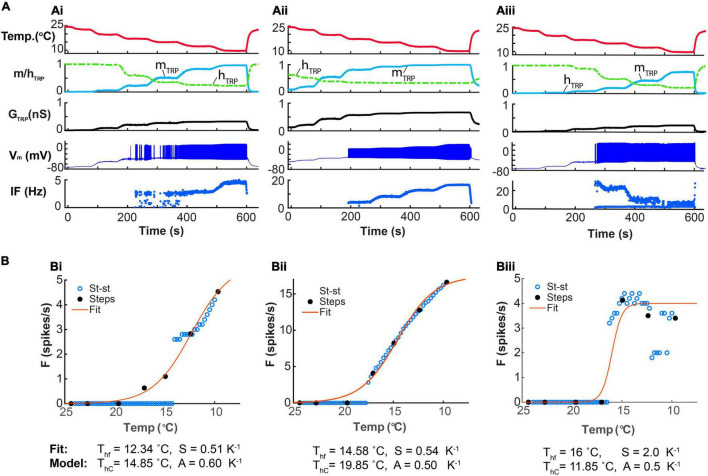
CIII neuron model responses to steady-state temperature. **(Ai–Aiii)** Graphs from the top to the bottom show the experimental temperature protocol with steps of temperature changes, TRP activation and inactivation over time, instantaneous TRP conductance over time, electrical activity over time, and the spiking rate over time. **(Bi–Biii)** Model temperature-response curves based on experimental step-stimulation protocol (black-closed circles). These curves were fitted with Boltzmann function (the red line) with the following temperature of half activation and steepness: 12.34^o^C, 0.51 K^–1^ **(Bi)**; 14.58^o^C, 0.54 K^–1^ **(Bii)** and 16^o^C, 2. K^–1^ **(Biii)**. Model temperature-response curves obtained at various constant steady-state temperatures are shown as blue open circles. Parameters for **(Ai,Bi)**: $\bar{G_{TRP}}$ = 1.5 nS, A = 0.6 K^–1^, *N* = 5, *T*_*h*_ = 288 K, Ca_*h*_ = 900 nM, τ_*hTRP*_ = 5 s, τ_*mTRP*_ = 0.002 s. Parameters for **(Aii,Bii)**: $\bar{G_{TRP}}$ = 2 nS, A = 0.5 K^–1^, *N* = 1, *T*_*h*_ = 293 K, Ca_*h*_ = 900 nM, τ_*hTRP*_ = 5 s, τ_*mTRP*_= 0.002 s. Parameters for **(Aiii,Biii)**: $\bar{G_{TRP}}$ = 1.5 nS, A = 0.5 K^–1^, *N* = 5, *T*_*h*_ = 285 K, Ca_*h*_ = 700 nM, τ_*hTRP*_ = 10 s, τ_*mTRP*_ = 0.002 s.

Biological CIII neurons have different types of responses to steady-state cold temperature magnitude– sigmoidal and non-sigmoidal (bell-shaped) temperature-response curves (Figure 4Aii). To examine model responses to steady-state temperature, we applied an experimental step-stimulation protocol. Such temperature protocol introduced a slow, quasi-steady-state change of temperature to our model (Figures 12Ai–iii). In addition to the step-stimulation protocol, we recorded steady-state spiking rates of the model at different constant temperatures in the range 24…10^o^C. We plotted relationships of spiking rates over temperature for both temperature protocols (Figures 12Bi–iii).

We demonstrated three examples of models distinct in parameters, determining TRP activation and inactivation. These models produced different types of electrical activity and steady-state dependencies on temperature (Figure 12). The example in Figure 12Ai showed that activation and inactivation followed steps of temperature, and TRP conductance is increasing with temperature decrease (Figure 12Ai). Fitted with the Boltzmann function, the dose-response curve demonstrated a relatively high threshold for activation, *T*_hC_ = 12.3^o^C (Figure 12Bi). An example in Figure 12Aii had similar behavior of activation, inactivation, and instantaneous conductance, but all these characteristics underwent saturation earlier than in Figure 12Ai because of the low threshold (higher temperature of half activation, *T*_hC_ = 19.85^o^C). It is interesting to note that, while activation and inactivation in Figure 12Aii underwent saturation, the spiking rate kept growing with temperature decreasing. Another example (Figure 12Aiii) demonstrated that, at a certain parameter set for TRP current activation and inactivation, the spiking rate could have extremum and decline with lover temperatures, whereas instantaneous TRP conductance increases with a temperature drop (Figure 12Aiii).

The model responses (Figures 12Ai–iii) were classified by different types of temperature-response curves: sigmoidal type (Figures 12Bi,ii) and non-sigmoidal type of dependence with a maximum firing rate at a certain temperature (∼15^o^C) (Figure 12Biii). These temperature-response curves were different in the temperature half activation and the steepness (sensitivity). These types of model temperature-response curves qualitatively corresponded to what we observed in experimental recordings in response to the step-stimulation temperature protocol (Figure 4Aii).

Thus, the type of the temperature-response curve can be determined by parameters of TRP current, and it does not directly correspond to the passage of conductance of TRP current. It is, instead, determined by the combination of kinetic parameters of TRP current and their state evolution. Various combinations of parameters for TRP activation and inactivation may reflect the variability in the expression of TRP channels and/or their isoforms among individual CIII neurons.

In summary, based on the kinetics of temperature-dependent activation and Ca^2+^-dependent inactivation of TRP current, our model explains the following mechanisms: (1) generation of the pronounced peak of the spiking rate, which was dependent on the rate of temperature change; (2) CIII response after adaptation that was dependent on the cold temperature magnitude (3) the cell-to-cell variability of the time constants of decay at fast temperature change; (4) the character of the temperature-response curve; (5) variability in the temperature of half activation of the spiking rate, time constant of spike frequency decay, peak, and steady-state frequency. Such model interpretation allowed us to explain different types of temperature coding: the rate of temperature change with subsequent frequency adaptation and coding of the magnitude of steady thermal stimuli.

## Discussion

We investigated spiking responses of *Drosophila* CIII neurons to transient and steady noxious cold temperature stimuli. CIII neurons are primary afferents that tile the epidermis of *Drosophila* larvae (Grueber et al., 2002). Using extracellular electrophysiological recording from these neurons, we described the basic spiking properties of CIII neurons, which allow them to encode both the temperature in the cold range and the rate of temperature change. We also developed the biophysical conductance-based family of CIII models to infer membrane dynamics underlying their cold-evoked responses. Based on computational modeling, we showed that kinetics of TRP currents offer a mechanism, explaining experimental phasic-tonic responses of CIII neurons, coding of the rate of temperature change and cold temperature magnitude, the character of the temperature-response curve, as well as variability in CIII cold-evoked responses.

Both flies and mammals have a population of temperature-sensitive neurons, which detect the direction of temperature change, *i.e.*, temperature decrease for cold-sensing and increase for hot-sensing cells, and whose activity levels increase with the magnitude of temperature change (Gallio et al., 2011; Turner et al., 2016). In general, cold-sensing afferents of different species can be categorized into two types–those who sense the temperature in an innocuous range and those who detect noxious thermal signals (McKemy, 2007, 2013). The first type is classified as innocuous cold-sensing neurons, which usually have spontaneous activity at normal skin temperature and increase their firing rate when temperature decreases. When the temperature rises, their spiking rate decreases or drops to zero (Gallio et al., 2011; Olivares et al., 2015). These low threshold primary afferents are activated at cool temperatures (near 30^o^C in mammals), which do not cause tissue damage and are not perceived as pain (McKemy, 2013; Waldman, 2016). The second type is cold nociceptors, which are silent at normal temperatures and are activated at the noxious cold temperature roughly below 15^o^C. The innocuous cold-sensitive neurons have a transient response and rapid frequency adaptation to non-noxious thermal stimuli, whereas the cold nociceptors have sustained action potential firing and limited adaptation to noxious cold temperatures (Zimmermann et al., 2009). Interestingly, cold-sensitive afferents of both mammals and insects can be multimodal. For example, mammalian DRG neurons are activated with both cold temperature and mechanical stimuli (Kumazawa, 1996; Zimmermann et al., 2009; Prescott and Ratte, 2012; Prescott et al., 2014; Wang et al., 2018); *Drosophila* CIII neurons are activated by noxious cold and innocuous mechanical stimuli (Yan et al., 2013; Turner et al., 2016, 2018). Thus, the anatomical, structural, and molecular aspects of temperature-sensitive neurons are consistent across species, suggesting that insects and mammalians could have similar principles of temperature coding at the peripheral levels (McKemy, 2007; Gallio et al., 2011). We considered two subtypes of CIII neurons, ddaA and ddaF, which were detected by a genetic marker and distinguished by morphology and position. Both types were usually silent or had spontaneous low-frequency spiking at ambient temperature; they both responded to a fast temperature drop with an initial peak of the spiking rate at a fast temperature decline, which was followed by a subsequent frequency adaptation that brought the spiking rate down to a steady state. The spiking rate of the initial peak and the subsequent steady state both depended on the temperature of the cold stimulus; the lower the temperature of the stimulus, the higher the spiking rate of spikes in the response. A similar correlation between the spiking rate and the temperature drop has been reported in cold-sensitive neurons of other animals, including mammals and insects (Loftus, 1968; Merivee et al., 2003; Gingl et al., 2005; Vriens et al., 2014; Nagel and Kleineidam, 2015). There was no statistical difference in cold-evoked spiking responses between ddaA and ddaF neurons.

### Cell-to-Cell Variability in Temperature Sensitivity Across CIII Neurons May Indicate Population Coding

We found that there is high variability in temperature of half activation of the spiking rate (ranging between 11 and 25°C) and steepness (ranging from between 0.22 and 2.59) across individual CIII neurons. This may suggest that individual CIII neurons are distinctly tuned to a specific temperature range and have different sensitivity to temperature. Such diverse sensitivities may allow CIII neurons to detect not only nociceptive cold but also temperatures in the innocuous cold range. Similar cell-to-cell variability in the magnitude of cold-induced responses was observed in both mammals (Ran et al., 2016; Wang et al., 2018) and insects (Loftus, 1968; Merivee et al., 2003).

In addition, the fact that CIII neurons are also innocuous mechanosensors (Yan et al., 2013; Turner et al., 2016, 2018) could also explain high cell-to-cell variability. Although the level of intracellular calcium in response to noxious cold was significantly higher than gentle-touch-evoked Ca^2+^ response (Turner et al., 2016), gentle mechanical stimuli elicit discharge with a burst of action potentials, followed by very rapid spiking rate decay up to zero at a steady-state stimulus (Yan et al., 2013). This may indicate that the mechanical disturbance during recording might have been attributed to the cell-to-cell variability among CIII neurons. However, since similar large variations in the temperature of half activation of the spiking rate were observed even in experiments with the slow ramp and step-stimulation protocols that were performed without solution switching, it is unlikely that the variation is due to mechanical perturbance by solution switching.

### CIII Neurons Could Encode at Least Three Temperature Characteristics: The Rate of Temperature Change, the Magnitude of Steady Temperature, and the Direction of Temperature Change

Electrophysiological recordings revealed that the majority of CIII neurons have a phasic-tonic response to noxious cold temperature stimulation. They strongly responded to a fast temperature drop with a pronounced peak of the firing rate (phasic response), which was followed by a subsequent frequency adaptation when the temperature reached a steady-state level (tonic response). The proportion of neurons showing a peak response increased with the increasing amplitude of the cold stimulus. At a fast temperature drop down to 10°C, approximately 70% of CIII neurons showed a peak in the spiking rate. Similar to CIII neurons, phasic-tonic responses to cold temperature have been observed in cold-sensitive neurons of various species, including insects and mammals: ants (Ruchty et al., 2009, 2010; Nagel and Kleineidam, 2015); cockroaches (Loftus, 1968; Nishikawa et al., 2004); mosquitoes (Davis and Sokolove, 1975; Gingl et al., 2005); cricket (Nishikawa et al., 1985); ground beetle (Merivee et al., 2003); tick (Hess and Loftus, 1984; Ameismeier and Loftus, 1988; Must et al., 2006); mammals: mice (Zimmermann et al., 2009; Parra et al., 2010; Olivares et al., 2015; Ran et al., 2016), rats (Braun et al., 1999), and cats (Duclaux et al., 1980; Schäfer et al., 1982).

Phasic-tonic responses are characterized by frequency adaptation to a steady-state level. Along with temperature sensation, neural adaptation is often seen in various sensory systems of both vertebrates and invertebrates (Kaissling et al., 1987; Rieke and Rudd, 2009; Ruchty et al., 2010). Adaptation enables sensory systems to adjust the gain to particular aspects of the stimulus (Chance et al., 2002). For example, adaptation serves as a mechanism for gain control of thermoreceptor neurons (Tichy et al., 2008). Neural adaptation enables neurons to increase the working range of the sensor in which changes in stimulus can be detected (Kaissling et al., 1987; Rieke and Rudd, 2009; Ruchty et al., 2010). This property is especially important for such tiny insects as *Drosophila* larva, for which temperature conditions have a crucial impact (Himmel et al., 2021).

Among the cold-sensitive neurons, there are many examples across species that have been reported to produce a phasic-tonic response to the rapid temperature drop, where the cold-evoked firing rate or calcium signal increased with the magnitude of a temperature drop, ΔT: insects (Loftus, 1968; Davis and Sokolove, 1975; Merivee et al., 2003; Nishikawa et al., 2004; Gingl et al., 2005; Must et al., 2006; Ruchty et al., 2009; Frank et al., 2015) and mammals (Craig et al., 2001; Ran et al., 2016; Wang et al., 2018). The mammalian cold-sensitive neurons were reported to respond to temperature changes regardless of the cooling rate (Craig et al., 2001; Ran et al., 2016; Wang et al., 2018). The rate of cooling (the rate of temperature change, dT/dt) is another important characteristic of thermal stimulus. Neurons of different species whose firing rate increases with the rate of temperature change are represented in the following studies: insects (Loftus, 1968; Davis and Sokolove, 1975; Tichy, 1979; Ameismeier and Loftus, 1988; Gingl et al., 2005; Tichy et al., 2008; Ruchty et al., 2010; Nagel and Kleineidam, 2015); and mammals (Olivares et al., 2015).

We revealed that the maximal firing rate at the phasic component of CIII responses has a significant correlation with a rate of temperature change. More neurons exhibited a pronounced peak of the firing rate immediately after the onset of stimulation in response to fast temperature drops. In contrast, in response to a slow temperature decrease, most CIII neurons showed a gradual increase in the spiking rate that reached a maximum at various times, while a few neurons showed a fluctuating increase with clusters of spikes appearing several times during the temperature downslope. Collectively, these findings suggest that CIII neurons are equipped to encode the rate of temperature cooling. Rapid changes of temperature trigger stereotyped protective behavioral responses: avoidance of potentially harmful stimuli, reducing the exposed body surface area or rapid cold hardening in ectotherms (Lee et al., 1987; Burton et al., 2016; Turner et al., 2016). In contrast, a slow temperature change does not typically cause immediate behavioral response (Yarmolinsky et al., 2016). Slow and long-term temperature change promotes cold acclimation (Waldman, 2016; Teets et al., 2020). It has been previously shown that extended exposure to noxious cold in *Drosophila* larva triggers cold acclimation, and that this is mediated by CIII nociceptive neuron activity (Himmel et al., 2021).

CIII neurons encode the rate of temperature change only in one direction – when the temperature decreases. When the temperature rose from noxious cold to room temperature, the spiking rate quickly declined back to the basal level (0–0.37 Hz), which was significantly lower than those during the temperature decrease. Suppression of electrical activity at a temperature rise is a common feature of cold-sensitive neurons observed in both insects and mammalians (Merivee et al., 2003; Olivares et al., 2015; Ran et al., 2016). Moreover, CIII’s firing at temperature increase was also dependent on the speed of a temperature rise. In cases of a fast temperature rise, most CIII neurons became silent, whereas the majority of neurons continued to spike when the temperature returned slowly back to room temperature.

When the temperature reaches a steady level after the temperature drops down, CIII responses turn to their tonic phase. Adapted CIII frequency does not drop to its basal level at room temperature but keeps constant. We found that the steady-state cold-evoked spiking rate of CIII neurons was dependent on the magnitude of cold temperature. The steady-state spiking rate increased with decreasing value of the cold temperature. Such property of CIII neurons is similar to cold-sensitive neurons of other species (Bade et al., 1979; Duclaux et al., 1980; Schäfer et al., 1982; Craig et al., 2001; Must et al., 2006; Frank et al., 2015; Nagel and Kleineidam, 2015; Alpert et al., 2020). For CIII neurons, we fitted graphs of the firing rate over temperature, i.e., temperature-response curves, with a Boltzmann function to calculate the temperature of half activation of the spiking rate and steepness of individual CIII neurons. These characteristics characterize their sensitivity to temperature. We found high variability in temperature of half activation of the spiking rate and steepness across individual CIII neurons, ranging between 11 and 25°C. Similar cell-to-cell variability in the magnitude of cold-induced responses was observed in other insects (Loftus, 1968; Merivee et al., 2003; Nagel and Kleineidam, 2015), as well as in mammals (Craig et al., 2001; Ran et al., 2016).

This high cell-to-cell variability of all described properties of CIII neurons suggests that population CIII neurons could encode the rate of temperature decrease. Other studies also report cold-sensitive neurons that encode both parameters of a thermal signal – the rate of temperature change and steady cold temperature (Loftus, 1968; Merivee et al., 2003; Nishikawa et al., 2004; Gingl et al., 2005; Tichy et al., 2008; Olivares et al., 2015). The phasic component of the CIII response (the peak of the firing rate) is dependent on the rate of the temperature drop. A tonic component appears when the firing rate stabilizes at a new steady cold temperature level, and it is dependent on the magnitude of cold temperature.

### The Dynamics of TRP Current Could Explain the Response of the CIII Neurons to the Rate of Temperature Change and Magnitude of Steady Cold Temperature

CIII neurons express a suite of TRP channels: Trpm, Pkd2, NompC, Brv1, and TRPA1, select members of which have been implicated in the cold-evoked behavior of *Drosophila* larva (Turner et al., 2016, 2018; Himmel and Cox, 2020). There is strong experimental evidence that *Drosophila* Pkd2 serves as a temperature sensor (Turner et al., 2016): Overexpression of Pkd2 in heat nociceptors, CIV neurons, confers cold sensitivity to these otherwise largely cold-insensitive neurons as measured by enhanced cold-evoked calcium response under decreasing temperatures. In addition, Pkd2 mutants exhibit a significant decline of cold-evoked calcium responses in CIII neurons (Turner et al., 2016). Due to a lack of experimental data on temperature-dependence of gating kinetics of *Drosophila* TRP channels, we used a computational approach, assuming that the basic functional properties of TRP channels are conserved across species (Venkatachalam and Montell, 2007; Falcon et al., 2019). Thus, our model uses a basic TRP current, combining properties of the TRP channels described for other species. In our computational model, the TRP current represents one of the implicated TRP currents or a current that lumps together all different temperature-dependent TRP currents expressed in CIII neurons.

Several biophysical models have been previously developed to investigate mechanisms of cold-temperature neural coding (Longtin and Hinzer, 1996; Braun et al., 1998, 2001; Huber et al., 2000; Finke et al., 2010). These models act as cold temperature sensors without TRP currents. They implicate the mechanism of thermosensation based on scaling factors for time constants and maximal conductances for non-TRP currents. Thus, due to temperature-evoked changes in time constants and maximal conductances of non-TRP currents, patterns of neural activity vary at different temperatures. In addition, the contribution of different non-TRP currents to activity patterns can be significantly changed at distinct temperatures (Korogod and Demianenko, 2017; Alonso and Marder, 2020). Mathematical models mentioned above reproduced cold-evoked activity patterns only at steady temperatures but did not explain transient spiking patterns at the temperature change, e.g., peaks of spiking rates. To explain the transient phase of the response of cold receptors, Olivares et al. (2015) developed a model of mouse cold-sensitive neurons innervating cornea with TRPM8 current incorporated into Huber and Braun’s model (Braun et al., 1998). As a result, the addition of TRP current with calcium-dependent desensitization to the cold receptor model enabled it to reproduce the dynamic (phasic) response evoked by a temperature change. Our model utilizes similar mechanisms to explain phasic responses of *Drosophila* CIII neurons to temperature changes. Unlike earlier cold receptor models (Longtin and Hinzer, 1996; Braun et al., 1998, 2001; Huber et al., 2000; Finke et al., 2010) that have spontaneous discharge at higher temperatures, our model is silent, whereas it has spiking response at colder temperatures due to activation of TRP current. In our model, complete elimination of TRP current leads to suppression of electrical activity at cold temperatures (Figure 11D1 at $\bar{G_{TRP}}=0nS$).

Using computational modeling, we suggest mechanisms explaining how TRP channels could utilize temporal coding of the cold temperature value and the rate of temperature change in cold-sensitive CIII neurons. To determine these mechanisms, we developed a biophysical Hodgkin-Huxley style computational model based on transcriptomic data from larval CIII neurons and patch-clamp data on gating characteristics of *Drosophila* Na^+^ and K^+^ channels obtained from the literature (Hardie, 1991; Wang et al., 2013). Investigation of activation and inactivation parameters of TRP current enabled us to understand mechanisms of transient and steady cold temperature coding. Considering that TRP channels have Ca^2+^-dependent inactivation (desensitization), which leads to adaptation of cell response (Nilius et al., 2005; Rohacs et al., 2005; Gordon-Shaag et al., 2008; McKemy, 2013; Hasan and Zhang, 2018; Korogod et al., 2020) and that temporal scale for TRP channel inactivation is of the similar order as a spiking rate adaptation of cold-sensitive neurons (Latorre et al., 2011; Olivares et al., 2015), we used in our model TRP current with temperature-dependent activation and Ca^2+^-dependent inactivation. We created a family of CIII models, varying parameters of TRP activation and inactivation and tested them on different temperature protocols that were consistent with those applied to biological neurons.

We demonstrated that the dynamics of activation and inactivation of TRP channels could explain properties of the dynamic (phasic) and static (tonic) responses of CIII neurons at fast temperature decrease. Our CIII neuron model responds with a peak of the spiking rate to fast temperature decline and with spiking rate relaxation to a steady-state level, similar to what we have observed for biological CIII responses. Inactivation of TRP current operating on a time scale of ∼3 to 20 s could be responsible for the initial peak of the spiking rate at fast temperature change, subsequent spiking rate decay when the temperature reaches a steady-state level, and the silence of the majority of CIII neurons when the temperature rises to the initial value. Temperature-dependent activation and Ca^2+^-dependent inactivation of TRP current provide mechanisms for coding the rate of temperature change and the value of cold temperature. Consistent with the biological CIII neurons, the CIII neuron model with basic kinetics of TRP current is sensitive to the rate of temperature decrease. The higher speed of a temperature drop, the higher the maximal instantaneous conductance of TRP current, and the larger the peak of the firing rate. In contrast, slow temperature change does not induce a peak of CIII model activity at the beginning of cold stimulation; the spiking rate follows TRP conductance, which is growing with temperature decline. The steady-state frequency of the CIII neuron model, in turn, does not depend on the rate of temperature change but increases with the magnitude of cold temperature value.

In this study, we focused on the roles of the temperature dependence and Ca^2+^-dependent inactivation processes as the major factors determining temperature coding in CIII neurons. We left other factors like dependence on the membrane potential (Nilius et al., 2005, 2011) and the initial activation by intracellular Ca^2+^ concentration of some of the reported temperature-sensitive TRP currents (Venglarik et al., 2004; Wang et al., 2008) as beyond the scope of this study, although there is evidence for their contribution. Such calcium-dependent activation, followed by desensitization, might also contribute to the peak of the firing rate at the initial temperature drop.

We demonstrated that the peak and steady-state value of the spiking rate could be regulated by the properties of TRP currents. The spiking rate peak is mostly affected by activation characteristics of the TRP current. Thus, the peak of frequency is larger with higher values of the steepness of TRP activation, the temperature of half activation, the time constant of the TRP inactivation, and smaller values of the time constant of TRP activation. Static CIII response is mostly tuned by TRP inactivation parameters. Hence, the steady-state spiking rate grows with a decrease in the steepness of TRP inactivation and has a non-monotonic dependence on, and calcium concentration of, half inactivation. Maximal TRP conductance, which reflects the expression level of TRP channels, affects both peak and steady-state spiking rates. In addition, various parameter sets for TRP activation and inactivation characteristics can represent variability in CIII responses between individual CIII cells, which can be determined by variability in the expression ensemble of different temperature-sensitive TRP channels.

With modeling, we could not exclude other factors and mechanisms contributing to variability of responses. Within the scopes of our model, the origin of this variability is not conceptually limited to differences in kinetic properties of a certain type of the TRP channel. It may also originate from variability in the relative contribution of distinct types of TRP channels that are involved in CIII cold sensation. Individual neurons may express different proportions of these distinct types of channels. Also, variability can be potentially explained by downstream processes and contribution of non-TRP channels, which we did not explore in this article.

Finally, applying an experimental step-stimulation temperature protocol with steps of the cold temperature of a different magnitude to our computational model, we obtained temperature-response curves similar to experimental ones. We found that the temperature-response dependence does not always directly follow the changes in conductance of TRP current with temperature. Temperature-response curves are determined by activation/inactivation characteristics of TRP current in a state-dependent fashion.

Using our computational model, we investigated different temperature coding mechanisms dependent on the kinetics of TRP current. Together, these results indicate that, with the phenomenological representation of a TRP current with temperature-dependent activation and Ca^2+^-dependent inactivation, the model qualitatively reproduced the key features of CIII responses, reflecting the rate of temperature change and the magnitude of temperature. In addition, our computational model brings insight that different gating characteristics of TRP current may finely tune CIII neurons to encode the rate of temperature change and a magnitude of cold temperature value.

### Significance

Understanding cellular mechanisms of how primary sensory afferents encode specific features of cold and noxious cold stimuli by specific patterns of neural activity has a broad implication in sensory neuroscience and clinical relevance concerning the mechanisms of sensory neuropathies. It would describe and classify the mechanisms of functional and pathological cold sensation and cold hypersensitivity and could lead to new therapeutic strategies for the treatment of sensory neuropathies, e.g., neuropathic pain, cold allodynia, and visceral hypersensitivity.

## Data Availability Statement

The raw data supporting the conclusions of this article will be made available by the authors, without undue reservation.

## Author Contributions

NM, AS, and GC wrote the first draft of the manuscript. NM and AS prepared the figures. NM and GC developed and analyzed the computational model. AS performed *in vitro* experiments and statistical analysis of electrophysiological data. All authors contributed to conception and design of the study, manuscript revision, read, and approved the submitted version and final version of the manuscript.

## Conflict of Interest

The authors declare that the research was conducted in the absence of any commercial or financial relationships that could be construed as a potential conflict of interest.

## Publisher’s Note

All claims expressed in this article are solely those of the authors and do not necessarily represent those of their affiliated organizations, or those of the publisher, the editors and the reviewers. Any product that may be evaluated in this article, or claim that may be made by its manufacturer, is not guaranteed or endorsed by the publisher.
