# Supplementary material for: Transient and Steady-State Properties of Drosophila Sensory Neurons Coding Noxious Cold Temperature
Source: Front Cell Neurosci. 2022 Jul 25;16:831803. doi: 10.3389/fncel.2022.831803 (PMC9358291; doi:10.3389/fncel.2022.831803)
Supplement: Supplementary file 1 [file Data_Sheet_1.PDF]

Table S1. Experimental data that was extracted from temperature-response curves obtained based on slow- and step-stimulation protocols and used as model parameters set: *a* – the maximal spiking rate; *S* – the steepness of the curve; T_{hf} – temperature of half-activation of spiking rate. The parameter sets obtained with the slow-stimulation protocol and step-stimulation protocol were named as two groups: group I and group II correspondingly.

Group I, slow-stimulation protocol parameters sets:

Parameters set #	a, spikes/s	S, K^{-1}	T_{hf}, °C
G1-1	1.046	-0.8993	11.928
G1-2	0.909	-0.5784	15.665
G1-3	0.705	-1.7699	15.746
G1-4	0.76	-0.8439	15.79
G1-5	1.085	-1.4085	15.811
G1-6	1.785	-0.6988	16.002
G1-7	0.86	-1.1111	16.169
G1-8	2.174	-0.4785	17.155
G1-9	2.213	-1.0341	17.175
G1-10	1.435	-0.6046	17.85
G1-11	2.195	-12.048	17.971
G1-12	1.014	-0.4593	18.038
G1-13	1.175	-0.5187	18.5
G1-14	1.138	-1.1976	18.588
G1-15	0.515	-4.2373	19
G1-16	0.886	-0.4587	20.187
G1-17	0.98	-2.0964	20.41
G1-18	3.183	-0.4472	20.411
G1-19	0.945	-2.3364	21.471
G1-20	2.376	-0.6169	22.98
G1-21	1.752	-11.364	23.904
G1-22	1.218	-10.417	24.059

Group II, step-stimulation protocol parameters sets:

Parameters set #	a, spikes/s	S, K^{-1}	T_{hf}, °C
G2-1	0.833	-2.7027	11.054

G2-2	1.077	-1.642	11.362
G2-3	1.217	-1.1468	12.09
G2-4	1	-0.4131	12.289
G2-5	0.4	-6.0606	12.368
G2-6	0.638	-0.8489	12.461
G2-7	1.8	-0.4409	12.652
G2-8	0.34	-0.8985	13.739
G2-9	0.874	-0.8834	15.857
G2-10	0.51	-1.7794	16.95
G2-11	0.455	-1.7825	17.597
G2-12	0.429	-2.0202	17.823
G2-13	0.2	-2.3585	18.096
G2-14	1.05	-1.0256	18.15
G2-15	0.371	-0.9881	18.488
G2-16	0.232	-0.657	18.976
G2-17	1.267	-0.8969	19.537
G2-18	1.931	-0.6575	19.77
G2-19	1.419	-0.9434	20.188
G2-20	0.95	-0.6177	20.977
G2-21	0.274	-0.5967	21.075
G2-22	2.046	-0.4456	21.297
G2-23	0.075	-12.3457	21.992
G2-24	2.185	-0.8857	22.009
G2-25	0.274	-2.5907	22.911
G2-26	0.554	-0.6887	23.611
G2-27	0.227	-0.5845	24.894

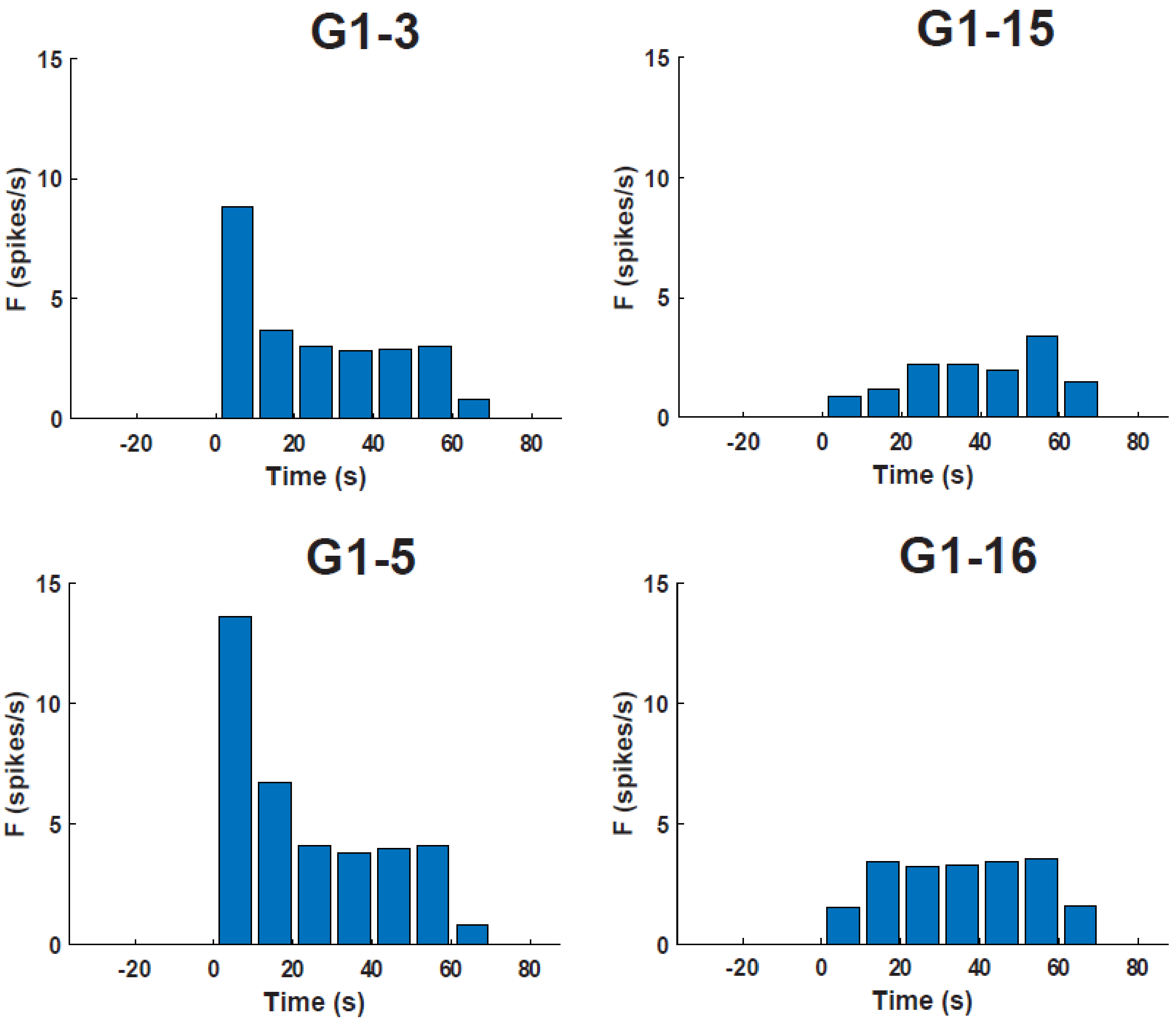

Figure S1. Representatives of model responses with different parameter sets based on slow-stimulation protocol. Numbers in the figure correspond to parameter set numbers from **Table S1**, group I. Depending on the parameter set model responded with a peak of spiking rate (sets G1-3 and G1-5) or without a peak (sets G1-15 and G1-16). Size of bin = 10 s.

Table S2. Model coefficients of variations for peak and steady-state when temperature protocol is fixed and parameters have variations (two first columns); parameters are fixed and temperature protocols have variations (two last columns). In the first two columns, every row corresponds to an experimental fast temperature stimulation trace when the temperature decreases to 10s. In the last two columns, every row corresponds to parameter sets.

Group I, slow-stimulation protocol parameters sets:

CoV of peak firing rate (**exp.parameters variations**)	CoV of steady-state firing rate (**exp.parameters variations**)	CoV of peak firing rate (**Temperature variations**)	CoV of steady-state firing rate (**Temperature variations**)
0.56	0.53	0.36	0.10
0.60	0.52	0.33	0.05
0.72	0.55	0.08	0.06
0.68	0.53	0.30	0.19
0.63	0.53	0.14	0.07
0.63	0.55	0.24	0.06
0.55	0.52	0.29	0.05
0.54	0.51	0.25	0.04
0.44	0.50	0.05	0.05
0.63	0.52	0.10	0.06
0.43	0.50	0.20	0.06
0.61	0.53	0.14	0.04
0.64	0.55	0.31	0.07
0.57	0.52	0.27	0.07
0.61	0.52	0.09	0.05
0.58	0.52	0.21	0.06
0.60	0.52	0.61	0.06
0.62	0.55	0.19	0.05
0.65	0.55	0.12	0.05
0.62	0.52	0.34	0.06
0.60	0.52	0.20	0.06
0.65	0.53	0.48	0.07

Group II, slow-stimulation protocol parameters sets:

CoV of peak firing rate (**exp.parameters variations**)	CoV of steady-state firing rate (**exp.parameters variations**)	CoV of peak firing rate (**Temperature variations**)	CoV of steady-state firing rate (**Temperature variations**)
0.64	0.45	0.36	0.29
0.84	0.45	0.12	0.09
0.81	0.46	0.15	0.05
0.68	0.45	0.23	0.07
0.73	0.45	0.25	0.10
0.71	0.44	0.25	0.17
0.71	0.43	0.34	0.14
0.72	0.47	0.03	0.06
0.77	0.45	0.05	0.03
0.78	0.45	0.25	0.14
0.78	0.46	0.05	0.04
0.86	0.44	0.19	0.10
0.65	0.45	0.15	0.05
0.75	0.45	0.04	0.07
0.72	0.45	0.18	0.03
0.77	0.45	0.37	0.32
0.64	0.44	0.38	0.10
0.86	0.45	0.26	0.09
0.77	0.45	0.42	0.15
0.45	0.45	0.51	0.11
0.50	0.48	0.31	0.10
0.74	0.45	0.26	0.08
0.70	0.45	0.03	0.05
0.72	0.45	0.35	0.07
0.69	0.45	0.28	0.14
0.71	0.45	0.20	0.09
